# Genomic Epidemiology and National Seroprevalence Reveal the Widespread Distribution of Palyam Viruses in China

**DOI:** 10.3390/v18060638

**Published:** 2026-06-03

**Authors:** Heng Yang, Wei Chen, Lei Xiao, Zhanhong Li, Lin Gao, Defang Liao, Jianbo Zhu, Xiao Wang, Huachun Li

**Affiliations:** 1 College of Agriculture and Life Sciences, Kunming University, Kunming 650214, China; yangheng2008.cool@163.com (H.Y.);; 2 Yunnan Tropical and Subtropical Animal Virus Diseases Laboratory, Yunnan Animal Science and Veterinary Institute, Kunming 650224, China; 3 Medical School, Kunming University of Science and Technology, Kunming 650500, China

**Keywords:** *Orbivirus*, Palyam virus, molecular epidemiology, seroprevalence

## Abstract

The taxonomic species Palyam virus (PALV) comprises a group of widely distributed, Culicoides-borne arboviruses linked to bovine reproductive disorders, yet its genetic diversity and seroprevalence in China have not been fully characterized. To address this gap, we obtained 29 PALV isolates from sentinel cattle in southern China, performed whole-genome sequencing on 15 representative strains, and conducted a nationwide serosurvey of 4660 cattle using a newly developed c-ELISA. These genomic data were integrated with global datasets for comprehensive phylogenetic and phylogeographic analyses. By establishing a global VP2-based framework, the Chinese PALV isolates were assigned to the Chuzan, Bunyip Creek, and D’Aguilar genogroups. In the global context, Chinese PALV strains exhibited the closest genetic affinity with strains from Japan, while phylogeographic reconstruction suggests at least two independent introductions from Japan during the 1980s and 1990s. Our survey revealed a high overall seroprevalence of 46.5% (95% CI: 44.7–47.5%) in cattle, demonstrating a pronounced latitudinal gradient with a sharp ecological threshold at 32.5° N. The virus is hyperendemic in humid southern China, with seroprevalence ranging from 33.0% to 88.8%, but attenuated in northern regions with seroprevalence less than 8.0%. These findings redefine PALV as a widespread “silent threat” in the East Asian arbovirus ecosystem, highlighting the need for coordinated transboundary surveillance.

## 1. Introduction

The genus *Orbivirus* (family *Sedoreoviridae*) comprises a diverse group of arboviruses, including well-characterized members such as Bluetongue virus (BTV), African horse sickness virus (AHSV), and Epizootic hemorrhagic disease virus (EHDV), which have caused substantial economic losses [1,2]. However, beyond these major pathogens, other lesser-studied members of the genus remain a potential threat to livestock health [3]. Multiple viruses belonging to the taxonomic species of Palyam virus (PALV) serve as prime examples of such neglected pathogens. Transmitted primarily by *Culicoides* biting midges, PALV is widely distributed across the tropical and subtropical regions of Asia, Africa, and Australia [4]. Although initially isolated from aborted bovine fetuses as early as the 1970s, the pathogenic potential of PALV remained unappreciated for over a decade. Its clinical significance was only established during a major outbreak of Chuzan virus (CHUV), one serotype of PALV, in Japan (1985–1986), which caused hydranencephaly–cerebellar hypoplasia (HCH) syndrome in more than 2463 calves [5]. Despite its severe historical impact, PALV remains profoundly understudied, a status that has hindered the development of effective diagnostic tools and thereby limited our understanding of the virus’s distribution, circulation dynamics, and pathogenicity.

The PALV genome comprises ten double-stranded RNA (dsRNA) segments (Seg-1 to Seg-10) that encode seven structural proteins (VP1–VP7) and three non-structural proteins (NS1–NS3) [6]. The outer capsid proteins, VP2 (Seg-2) and VP5 (Seg-6), exhibit the highest sequence variability among the viral proteins. Critically, VP2 serves as the principal determinant of neutralization specificity, thereby defining the viral serotype [6]. Historically, up to 13 PALV serotypes have been proposed: CHUV, Kasba virus (KSBV), Vellore virus (VELV), Abadina virus (ABAV), Gweru virus (GWEV), CSIRO Village virus (CVV), D’Aguilar virus (DAV), Palyam virus (PALV), Nyabira virus (NYAV), Marondera virus (MARV), Apies River virus (ARV), Bunyip Creek virus (BCV), and Petevo virus (PETV) [4]. Nevertheless, their definitive taxonomic boundaries remain uncertain, as some ostensibly distinct serotypes share VP2 sequence identities exceeding 98%, suggesting significant taxonomic redundancy. Given the lack of standardized reference viruses and antisera for PALV, establishing a VP2/Seg-2 sequence-based molecular framework is essential to define genogroups, resolve taxonomic redundancies, and harmonize global surveillance efforts.

In contrast to the hypervariable outer capsid proteins, the genomic segments encoding conserved core structural and non-structural proteins harbor informative phylogenetic signals and often retain phylogeographic structure [4,7]. Early comparative genomics analyses of isolates from India, Australia, and Africa resolved global PALV strains into two primary lineages: a distinct African clade and a broader assemblage encompassing strains from Asia and Australasia [4]. More recently, comprehensive genomic characterization of 15 Japanese isolates refined this spatial paradigm, proposing that viruses from Japan, China, and India constitute a shared Asian gene pool, punctuated by occasional reassortment with Australian lineages [7]. While these foundational studies provided preliminary insights, they were based on datasets in which Chinese sequences were either absent or significantly underrepresented, leaving the precise micro-evolutionary trajectories, transboundary dispersal routes, and segment-specific reassortment dynamics of PALV within East Asia completely uncharacterized.

As a pivotal component of the East Asian arbovirus ecosystem, China harbors immense domestic ruminant populations and occupies a critical geo-epidemiological position in regional arbovirus surveillance and preparedness [8]. However, the molecular and serological epidemiology of PALV in China remains highly fragmented and largely unmapped. CHUV was first isolated from asymptomatic sentinel cattle in Yunnan, China, in 2012 [9]. Subsequent isolations from cattle in Guangxi Province and yaks on the Qinghai Plateau [10,11] indicated that CHUV possesses an expansive geographical footprint across China. Our long-term sentinel surveillance in Yunnan and Guangdong provinces (2012–2020) yielded multiple isolates of CHUV, along with two additional PALV serotypes: BCV and DAV. However, these investigations were limited to virus isolation and partial sequencing of Seg-2 and Seg-7 [12]. Consequently, the chronic scarcity of complete, high-confidence whole-genome sequences for these Chinese isolates, juxtaposed with the absence of a large-scale serological survey, has profoundly obscured the true genetic architecture, evolutionary provenance, and comprehensive seroprevalence landscape of PALV within mainland China.

To address these knowledge gaps, we obtained 29 PALV isolates from sentinel cattle in Yunnan and Guangdong provinces between 2012 and 2020, and performed whole-genome sequencing on 15 representative strains identified as CHUV, BCV, and DAV. By integrating these newly sequenced genomes with global datasets, we aimed to definitively assign the molecular types of the Chinese isolates and to reconstruct the specific evolutionary trajectories, local reassortment dynamics, and transboundary dispersal pathways of PALV lineages within East Asia. Complementing this genomic characterization, we developed a group-specific competitive ELISA (c-ELISA) and conducted a comprehensive cross-sectional survey of 4660 cattle across 15 provinces between 2016 and 2018. Ultimately, this integrated approach delineated nationwide exposure patterns and their complex geospatial determinants, representing the first large-scale genomic and seroepidemiological assessment of the PALV circulated in mainland China.

## 2. Materials and Methods

### 2.1. Large-Scale Serum Sampling Across China

From 2016 to 2018, a nationwide cross-sectional serological survey was executed across 15 provinces and 119 counties in China. Geospatially, the sampling architecture intersected a wide geographic range (18.2° N–53.3° N; 73.6° E–126.0° E)and a steep altitudinal gradient from sea level up to 5000 m, effectively capturing multiple climate zones of China (Table 1). All samples were obtained from clinically healthy cattle (12–30 months of age). The sampled cattle had no history of PALV vaccination, as no commercial vaccine is currently available in China. The survey employed a four-stage sampling design to ensure coverage of major livestock farming zones and diverse climatic regions. (1) Province level: Fifteen provinces were purposively selected to represent China’s six major geographic and climatic regions. (2) County level: a total of 119 counties across 73 municipal-level divisions were identified to ensure broad geographic coverage. (3) Herd level: Within each selected county, representative cattle herds were identified, encompassing both intensive cattle farms and livestock trading markets. (4) Individual level: individual cattle within each herd were selected via simple random sampling based on unique ear tag numbers to minimize selection bias. For spatial analysis, a “sampling group” was defined as all samples collected from one county within a single calendar year. Ultimately, this sampling strategy yielded 129 distinct sampling groups (Table 1). A group was classified as PALV-positive if it contained at least one seropositive sample. Individual and group-level seroprevalence, and 95% Wilson confidence intervals (CIs) were calculated as described in Table S1.

### 2.2. Sentinel Animal Setting and Blood Sample Collection

Sentinel herds were established in Yunnan and Guangdong provinces for arbovirus isolation. Prior to enrollment, all sentinel animals (aged 6–12 months) were confirmed negative for both BTV nucleic acids and antibodies. Because the primary objective of the surveillance program was BTV isolation, any sentinel animal that seroconverted to BTV during the monitoring period was promptly replaced with a naïve animal to maintain the susceptibility and integrity of the herd. Between 2012 and 2017, five healthy native Yunnan Yellow calves were maintained as designated sentinel animals at each site in Shizhong, Jiangcheng, and Mangshi counties of Yunnan Province. Similarly, a sentinel group comprising five Holstein cows was established in Shantou City, Guangdong Province, in 2016. Furthermore, between 2019 and 2020, three water buffaloes (*Bubalus bubalis*) were introduced as sentinel animals for arbovirus isolation in Jinghong County, Yunnan Province. Detailed geographic and climatic characteristics of each surveillance site are provided in Table 2. During the annual peak arbovirus transmission season (1 May–30 October), heparinized blood, EDTA-anticoagulated blood, and serum samples were collected weekly from each animal for subsequent nucleic acid screening, isolation of arbovirus, and serological assays, respectively. All collected specimens were stored at 4 °C and transported to the laboratory within 48 h. Upon arrival, whole-blood samples were maintained at 4 °C, while sera were partitioned and stored at −20 °C until further analysis.

### 2.3. Development and Validation of a Competitive ELISA of PALV

To facilitate large-scale serological surveillance of PALV, a group-specific competitive ELISA(c-ELISA) was developed using purified CHUV (strain V144) as the diagnostic antigen. Briefly, virions propagated in *Aedes albopictus* (C6/36) cells were purified via ultracentrifugation (Section S1 of Text S1 in Supplementary Material). The morphological integrity and the purity of the virion were confirmed by negative-staining transmission electron microscopy (Figure S1). The total protein concentration of the purified virions was quantified using a BCA Protein Assay Kit (Takara Bio Inc., Shiga, Japan) and coated onto 96-well ELSIA microplates (100 μL/well) at an optimized coating concentration of 4.12 μg/mL (Section S3 of Text S1). Competitive polyclonal IgG was elicited in guinea pigs immunized with inactivated CHUV and purified using Protein G affinity chromatography (Section S2 of Text S1). A sequential competitive strategy was employed to ensure optimal sensitivity, in which test sera (1:20 dilution) were incubated before the addition of purified guinea pig anti-CHUV IgG (Sections S4 and S5 of Text S1). The presence of PALV-specific antibodies was detected using HRP-conjugated goat anti-guinea pig IgG (Abcam, Cambridge, MA, USA) and TMB substrate.

Percent Inhibition (PI) of the competitive ELISA (c-ELISA) was calculated using the formula: PI (%) = [1 − (OD_450_ of test sample/mean OD_450_ of negative controls)] × 100%. The diagnostic cutoff was determined via Receiver Operating Characteristic (ROC) analysis using a reference panel of 150 PALV-positive bovine sera (*n* = 50 each for CHUV, BCV, and DAV) and 100 PALV-negative bovine sera, all of which were confirmed by the virus neutralization test (VNT) [13]. Based on the maximum Youden index, the diagnostic threshold was set at PI ≥ 50.0%. Accordingly, samples with PI < 45.0% were considered negative, whereas those ranging from 45.0% to 50.0% were classified as equivocal and subjected to retesting. At this cutoff, the diagnostic sensitivity was 93.3% (95% CI: 90.5–97.2%) and the specificity was 97.3% (95% CI: 96.2–99.9%). To evaluate analytical specificity, a panel of positive bovine antisera (*n* = 15 each) against other bovine pathogens was evaluated, which included BTV, EHDV, Akabane virus (AKAV), bovine ephemeral fever virus (BEFV), and foot-and-mouth disease virus (FMDV). No cross-reactivity was observed, with all PI values consistently remaining below 20%. Assay reproducibility was confirmed by intra- and inter-assay coefficients of variation (CVs) of less than 8% and 10%, respectively.

### 2.4. Virus Isolation and Identification

To optimize diagnostic efficiency and avoid blind mass-screening of the entire routine sample inventory, the tracking of PALV infection in sentinel animals was driven by a retrospective, seroconversion-triggered sampling framework. The onset of seroconversion was defined as the first week in which of an animal’s serum tested positive by the group-specific c-ELISA for PALV described above. Heparinized blood and EDTA-anticoagulated blood samples collected exactly one week before the seroconversion were retrospectively selected for molecular verification and subsequent pathogen isolation. Total RNA from these selected EDTA whole-blood samples was extracted using a MagMAX Express-96 Deep Well Magnetic Particle Processor (Thermo Fisher Scientific, Waltham, MA, USA) according to the manufacturer’s protocol. Targeted confirmatory screening of PALV genomic RNA was performed via qRT-PCR assays targeting Seg-7 (group-specific) and Seg-2 (serotype-specific) using the specific primers and TaqMan probes outlined in Table S2 of the Supplementary Material.

For virus isolation, heparinized blood samples collected one week before seroconversion were retrospectively selected for virus isolation, according to our previously established arbovirus isolation protocol [14]. Briefly, virions were released from erythrocytes via lysis in sterile distilled water. The clarified supernatants were blind-passaged for 1 to 3 rounds in C6/36 cells, followed by 3 to 5 serial passages in Baby Hamster Kidney (BHK-21) cells. Isolates showing extensive cytopathic effects (CPE) were identified by group- and serotype-specific RT-PCR targeting Seg-7 and Seg-2 of PALV (Table S2) [12], respectively. Clonal virus stocks were obtained via a single round of plaque purification on BHK-21 cells using a 1% methylcellulose overlay and 0.01% neutral red staining [14].

### 2.5. Viral Genome Amplification and Next-Generation Sequencing

Based on preliminary sequence analysis for Seg-2 and Seg-7 of isolated PALV strains [12], 15 representative strains were prioritized for whole-genome sequencing. Following propagation in BHK-21 cells, total RNA was extracted from infected cells with 70% CPE using TRIzol Reagent. Viral dsRNA was enriched via differential 2 M lithium chloride (LiCl) precipitation (Sigma-Aldrich, St. Louis, MO, USA) and subsequently converted to cDNA employing the FLAC method with an anchor primer (5′p-GACCTCTGAGGATTCTAAAC/iSp9/TCCA GTTTGAATCC-OH-3′) [15]. Libraries were prepared with the TruSeq DNA Sample Prep Kit V2.0 (Illumina, Inc., San Diego, CA, USA) using purified FLAC RT-PCR amplicons and sequenced on the Illumina HiSeq 2500 platform (150-bp paired-end). Raw reads were quality-assessed using FastQC (v0.11.9) and trimmed/filtered with Trimmomatic (v0.39) [16]. Host-derived reads were removed by subtractive mapping against the *Mesocricetus auratus* genome (GCA_000349665.1) using Bowtie2 [17]. *De novo* assembly was performed using SPAdes (v3.15) with metaviralSPAdes [18], and the obtained contigs were assigned to PALV segments via BLASTn (v2.13.0). These segment sequences were iteratively refined through read remapping and comparative evaluation against PALV reference sequences to ensure both consensus accuracy and terminal integrity. Final consensus sequences (majority-rule) were generated using SAMtools (v1.10) [19]. To ensure high confidence in consensus calling, a stringent minimum per-site depth threshold of 2000× was enforced across all segments.

### 2.6. Phylogenetic and Evolutionary Phylogeographic Analyses

PALV sequences were retrieved from the National Center for Biotechnology Information (NCBI) Virus database (accessed 15 February 2025). Sequence inclusion was restricted to those featuring complete or near-complete coding regions (≥90% CDS coverage) and verifiable metadata (collection year and geographic origin). Genome segments were aligned using MAFFT (v7.037b) [20]. For each genomic segment, the best-fit nucleotide substitution model was automatically selected using ModelFinder integrated within IQ-TREE (v2.0) [21] based on the Bayesian Information Criterion (BIC). Maximum likelihood (ML) phylogenies were subsequently inferred under the identified best-fit models with 10,000 ultrafast bootstrap replicates. All alignments were screened for intragenic recombination in RDP5 [22] using seven detection methods (RDP, GENECONV, BootScan, MaxChi, Chimaera, SiScan, and 3Seq). Recombination events were accepted only when supported by at least four methods with Bonferroni-corrected *p*-values < 0.05.

The temporal signal for each segment of PALV was assessed through root-to-tip regression analysis in TempEst (v1.5.3) [23]. Segments with a coefficient of determination (R^2^) > 0.2 were considered suitable for time-scaled inference. The subsequent phylogenies were inferred using BEAST (v1.10.4) [24]. To determine the optimal evolutionary model parameters, a rigorous model selection process was performed on Seg-3, which exhibited the most robust temporal signal (R^2^ = 0.62). Six candidate combinations, comprising two molecular clock models (strict and uncorrelated lognormal relaxed [UCLN] clocks) paired with three coalescent priors (constant-size, exponential-growth, or Bayesian skyline) [25], were compared via path sampling (PS) and stepping stone (SS) marginal likelihood estimation [26]. These estimations were performed using 20 steps with 5,000,000 generations per step. The combination of a UCLN clock and a Bayesian skyline prior yielded the highest marginal likelihood (Table S3). Given that all PALV segments, except Seg-2 and Seg-6, shared evolutionary constraints, this optimized modeling framework was applied to all remaining segments. To ensure accuracy, substitution rates were estimated independently for each segment using uninformative priors.

To ensure that all parameters achieved an effective sample size (ESS) exceeding 200, three independent Markov chain Monte Carlo (MCMC) chains were run for 100 million generations, with a sampling frequency of every 10,000 generations. Log files and tree files from these independent runs were integrated using LogCombiner (v1.10.4), with the initial 10% of each chain discarded as burn-in. Convergence and mixing of the integrated results were assessed in Tracer (v1.7.2), with the detailed MCMC diagnostic metrics summarized in Table S4. The merged tree file was subsequently summarized as a maximum clade credibility (MCC) tree in TreeAnnotator (v1.10.4). To reconstruct the spatial diffusion history of PALV, discrete phylogeographic inference was conducted for Seg-3 across five designated geographic states: China, Japan, India, Australia, and Africa. This process employed a discrete-trait continuous-time Markov chain (CTMC) model incorporating asymmetric transition rates, with Bayesian stochastic search variable selection (BSSVS) [27] applied to identify significant migration pathways under default priors. Phylogenetic trees and MCC trees were visualized using FigTree (v1.4.4) and iTOL (Interactive Tree of Life).

### 2.7. Statistical Analyses

Statistical analyses were performed using GraphPad Prism (v9.0) and IBM SPSS Statistics (v29.0). Seroprevalence rates were calculated with 95% Wilson confidence intervals (CIs), excluding samples with equivocal results. To quantify provincial-level differences, a mixed-effects logistic regression model was fitted, incorporating province and year as fixed effects and sampling group (county-year) as a random intercept. Adjusted odds ratios (aORs) were calculated relative to Inner Mongolia (reference category). For latitudinal trend analysis, sampling groups were aggregated into 1-degree latitude bins (retaining bins with >10 samples). To capture potential non-linear ecological threshold effects, a Generalized Additive Model (GAM) with penalized regression splines was employed to assess the relationship between bin-level seroprevalence and latitude. Model fitting and smoothing parameter optimization were performed to ensure robust trend visualization. Statistical significance was defined as a two-tailed *p*-value < 0.05.

## 3. Results

### 3.1. Geospatial Distribution and Multivariable Risk Analysis of National PALV Seroprevalence

**Figure 1:**
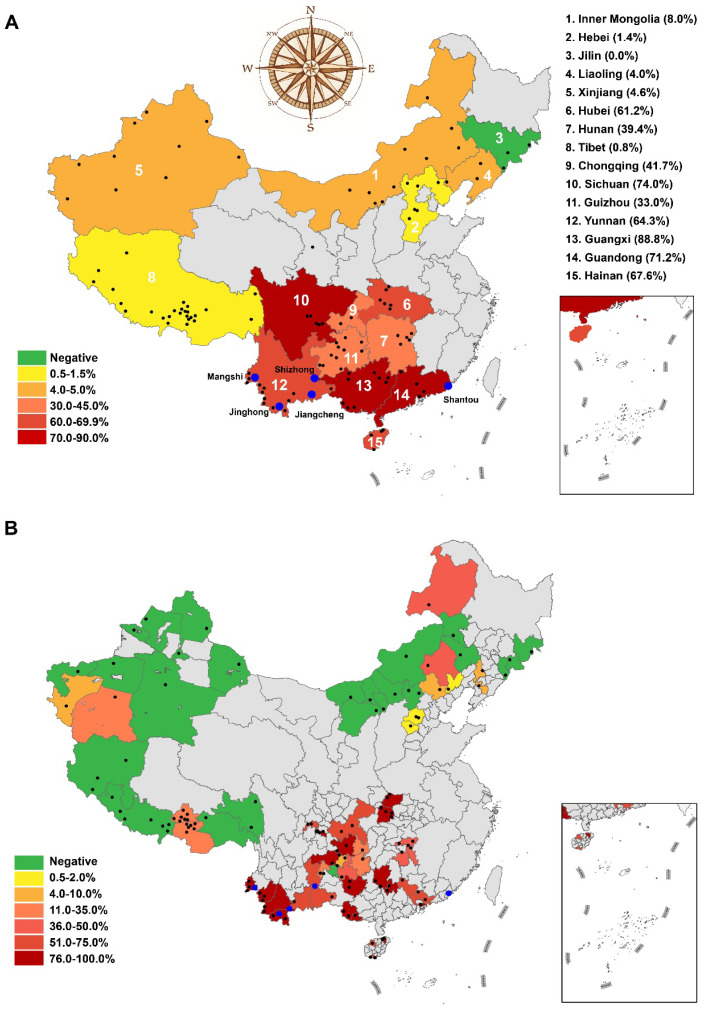
Geospatial distribution and multi-level seroprevalence of PALV in China, 2016–2018, revealing a pronounced latitudinal divide. Serosurvey data are visualized at the (**A**) provincial and (**B**) prefectural levels to illustrate the broad endemicity and regional hotspots of PALV. In both maps, regions are colored based on their respective seropositivity rates (see color scales), while non-surveyed regions are shown in gray. Surveyed counties (*n* = 119) are demarcated with black dots, and the five sentinel surveillance sites in Yunnan and Guangdong provinces are marked with blue dots. Map (**A**) provides the mean seroprevalence for each of the 15 surveyed provinces (numbered 1–15). Map lines delineate study areas and do not necessarily depict accepted national boundaries.

Between 2016 and 2018, we conducted a nationwide serological survey of PALV across 15 provinces in China, collecting a total of 4660 serum samples, of which 2167 tested positive (Table 3). The survey revealed a substantial overall PALV seroprevalence of 46.5% (95% CI: 44.7–47.5%), with seropositivity observed in all surveyed provinces except for Jilin (Table 4, Figure 1). In Northern China, PALV transmission was markedly attenuated, with seroprevalence rates ranging from a minimum of 1.4% (95% CI: 0.5–3.5%) in Hebei to a peak of 8.0% (95% CI: 3.5–15.0%) in Inner Mongolia. In sharp contrast, the virus was hyperendemic throughout Central and Southern China, where seroprevalence rates in nine provinces exceeded 30%, peaking at 88.8% (95% CI: 82.9–92.9%) in Guangxi (Table 4, Figure 1). This high-intensity circulation was predominantly concentrated in humid subtropical and tropical monsoon regions, where elevations generally remained below 1500 m. A conspicuous outlier was Tibet, which, despite its southwestern location, exhibited a minimal seroprevalence of 0.8% (95% CI: 0.2–3.0%), a phenomenon likely attributable to the high altitude (3000–5000 m) and extreme plateau alpine climate characterizing this region (Table 1).

To quantify these substantial regional differences in transmission risk, a multivariable logistic regression analysis was performed. Inner Mongolia was selected as the reference group because it represented the highest level of viral circulation among the northern pastoral regions, thereby providing a conservative baseline for comparison. Compared to this reference, significantly lower risks were observed in other northern provinces, particularly Jilin and Hebei, with adjusted odds ratios (aORs) of 0.1 (95% CI: 0.0–0.1) and 0.2 (95% CI: 0.1–0.5), respectively. Conversely, risk levels were markedly elevated across Central and Southern China (Table 4), with all aORs exceeding 10.0 (*p* < 0.001). This stark geographic disparity was exemplified by Hubei in Central China (aOR = 17.0; 95% CI: 8.6–38.4) and culminated in a peak risk in Guangxi (aOR = 89.5, 95% CI: 37.9–220.0).

**Figure 2:**
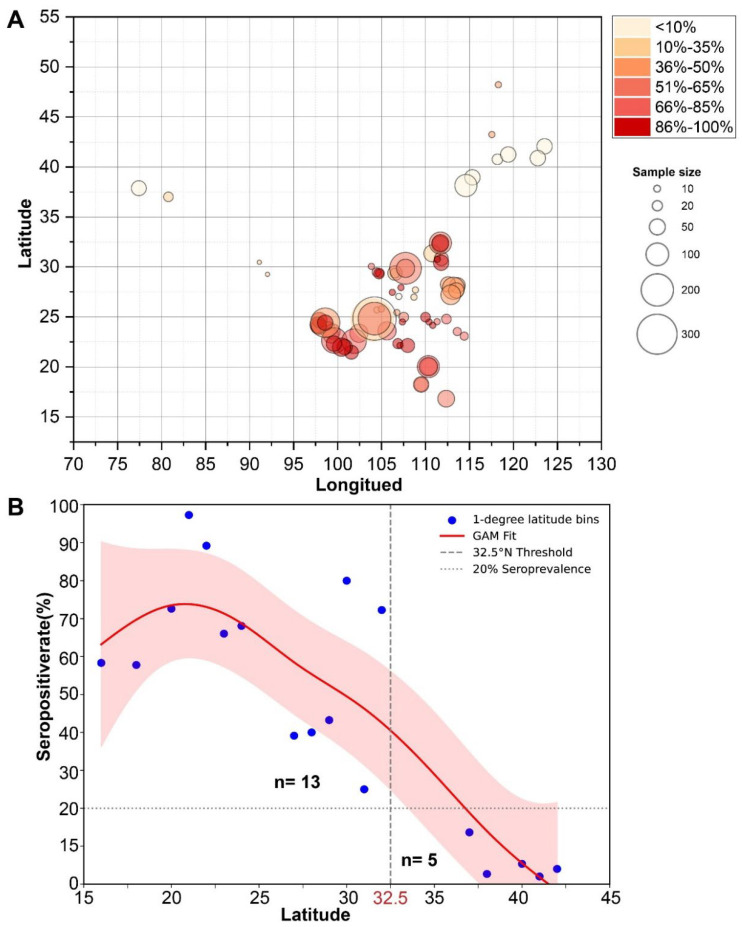
Geographical distribution and latitudinal dependence of PALV seroprevalence in China. (**A**) Geographical distribution of 78 seropositive sampling groups. Bubble color corresponds to the seroprevalence rate, and bubble size is proportional to the sample size. (**B**) Generalized Additive Model (GAM) and a Logistic Regression of seroprevalence against latitude. Each blue dot represents the seroprevalence for a 1-degree latitude bin. The red line shows the best-fit curve from a second-order polynomial regression, with the shaded area indicating the 95% CI. A latitudinal threshold is observed near 32.5° N (vertical dashed line). South of this latitude, 13 data points (*n* = 13) show seroprevalence predominantly above 20%, whereas all 5 data points (*n* = 5) to the north fall below this 20% level (horizontal dashed line).

To further delineate the spatial architecture of PALV transmission, the relationship between seroprevalence and geographic coordinates was analyzed for the sampling groups. Of the 129 sampling groups defined in this study, 78 were seropositive. The geographic distribution and prevalence intensity of these groups are visualized in a georeferenced bubble plot (Figure 2A). Georeferenced spatial analysis demarcated a high-risk endemic corridor (defined as >20% seroprevalence) largely constrained within a distinct geographic window between latitudes 17.5° N–32.5° N and longitudes 97.5° E–115° E. Notably, this corridor corresponds geographically to regions of high humidity and moderate-to-low elevation, primarily characterized by subtropical and tropical monsoon climates [28,29,30]. Furthermore, while an initial significant negative correlation (Spearman’s ρ = −0.67, *p* < 0.001) was observed between group-level seroprevalence and latitude, subsequent GAM analysis revealed a distinct non-linear dynamic underlying this latitudinal gradient (Figure 2B). The GAM curve statistically validated a sharp ecological threshold at approximately 32.5° N. South of this latitude, seroprevalence is maintained at hyperendemic levels; however, transmission risk abruptly attenuates in the northern regions. Importantly, this latitudinal model effectively explains the ecological trends in low-elevation regions, but it is bounded by the unique topography of Tibet. In this region, extreme vertical elevation acts as a primary barrier that supersedes latitudinal influences.

### 3.2. Co-Circulation of Three PALV Serotypes in Southern China

Between 2012 and 2020, a total of 2496 blood samples were collected from sentinel herds across five surveillance sites in Yunnan and Guangdong Provinces, yielding 29 PALV isolates (Table 3). Serotype-specific RT-PCR identified the 29 PALV isolates as CHUV (*n* = 17), BCV (*n* = 7), and DAV (*n* = 5). Most isolates were obtained in Yunnan (24/29, 82.8%), where CHUV, BCV, and DAV were detected, whereas Guangdong yielded five isolates (BCV, *n* = 4 and DAV, *n* = 1). CHUV was the most frequently detected serotype and was identified at all four Yunnan sentinel sites (Shizhong, Jiangcheng, Mangshi, and Jinghong). Shizhong County accounted for 75.0% (18/24) of the Yunnan isolates, including CHUV (*n* = 14), BCV (*n* = 2), and DAV (*n* = 2). The isolation of PALV strains exhibited a distinct seasonal trend, with 72.4% (21/29) of the strains isolated during June to September.

### 3.3. Whole-Genome Sequencing of Chinese PALV Isolates

As our preliminary Seg-2 and Seg-7 screening of the 29 PALV isolates revealed high genetic homogeneity (sequence similarity > 99.0%) among the same serotype strains isolated from the same year and location. To elucidate the genomic landscape of PALV in southern China, we conducted whole-genome sequencing on 15 representative isolates (Table 5) obtained from Yunnan and Guangdong provinces. Following quality filtering, the resulting clean reads, ranging from 0.67 to 2.10 Gb per isolate, were *de novo* assembled, yielding the complete coding sequences for all ten genome segments of each isolate. The sequencing provided robust coverage, with a mean mapping depth exceeding 8000× (ranging from 8500× to 38,000×) across the ten segments of each isolate. Per-isolate sequencing QC metrics (raw/clean bases, Q20/Q30, and GC%) are summarized in Table S5. Regarding within-sample variation, although minor single-nucleotide polymorphisms (SNPs) were detected at certain genomic positions, these minor variants typically represented less than 5% of the total reads at those specific sites. To maintain the genetic integrity of the dominant viral population, a majority-rule consensus calling strategy was strictly employed to determine the final sequences, which guaranteed maximum sequence accuracy across all segments, particularly at the segment terminals. The complete genome sequences of these 15 PALV isolates are available in GenBank under the accession numbers provided in Table 5.

### 3.4. Establishing VP2 and VP5 Phylogenetic Framework for the Precise Molecular Typing of Chinese PALV Isolates

**Figure 3:**
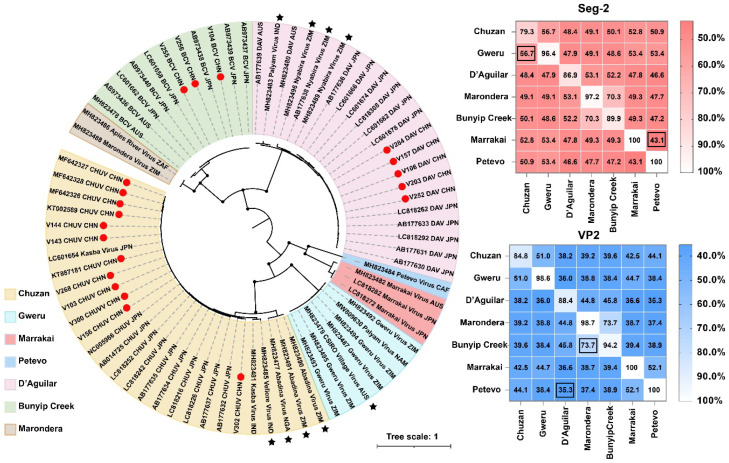
Global phylogenetic analysis of Seg-2/VP2 sequences and proposed sequence-based genogroup framework for Palyam virus. (**Left**): A maximum-likelihood (ML) phylogenetic tree was constructed from VP2 coding sequences. Nodal support ≥ 90% is indicated by solid black circles. Strains are labeled as ‘Accession number_Virus name_Country code’. The seven proposed genogroups of PALV in this study are highlighted by colored backgrounds. Chinese isolates are marked with red circles, and reclassified historical serotypes with black stars. (**Right**): Heatmaps display pairwise sequence identities. The upper matrix shows nucleotide identities (Seg-2); the lower shows amino acid identities (VP2). Diagonal values represent minimum intra-genogroup identities; off-diagonal values represent maximum inter-serotype identities. The black boxes in the heatmap respectively indicate the maximum and minimum values of similarity.

To definitively assign the serotypes of the 15 newly isolated Chinese PALV strains, we first constructed a phylogenetic reference framework based on global VP2 sequences. Phylogenetic analysis revealed that global VP2 sequences segregated into 7 well-supported monophyletic genogroups (Figure 3) with bootstrap values exceeding 90% at the key nodes. Though incorporating identity heatmap analysis, we established a VP2 amino acid identity threshold of 73.7% as a robust boundary for molecular typing of PALV (Figure 3). Utilizing these molecular criteria, the 15 Chinese isolates from the present study were unambiguously assigned to three major genogroups: Chuzan, Bunyip Creek, and D’Aguilar (Figure 3, Table 6). Notably, the Chinese isolates within each of the three genogroups consistently co-clustered with their corresponding Japanese strains, with intra-genogroup pairwise homologies exceeding 94.2% at the nucleotide level and 95.4% at the amino acid level. Furthermore, the phylogenetic analysis of VP5 (Figure S2) exhibited a clustering pattern highly concordant with the VP2 framework, providing additional support for these typing results.

### 3.5. Phylogenetic Analysis of the Eight Internal Genomic Segments of PALV Strains

**Figure 4:**
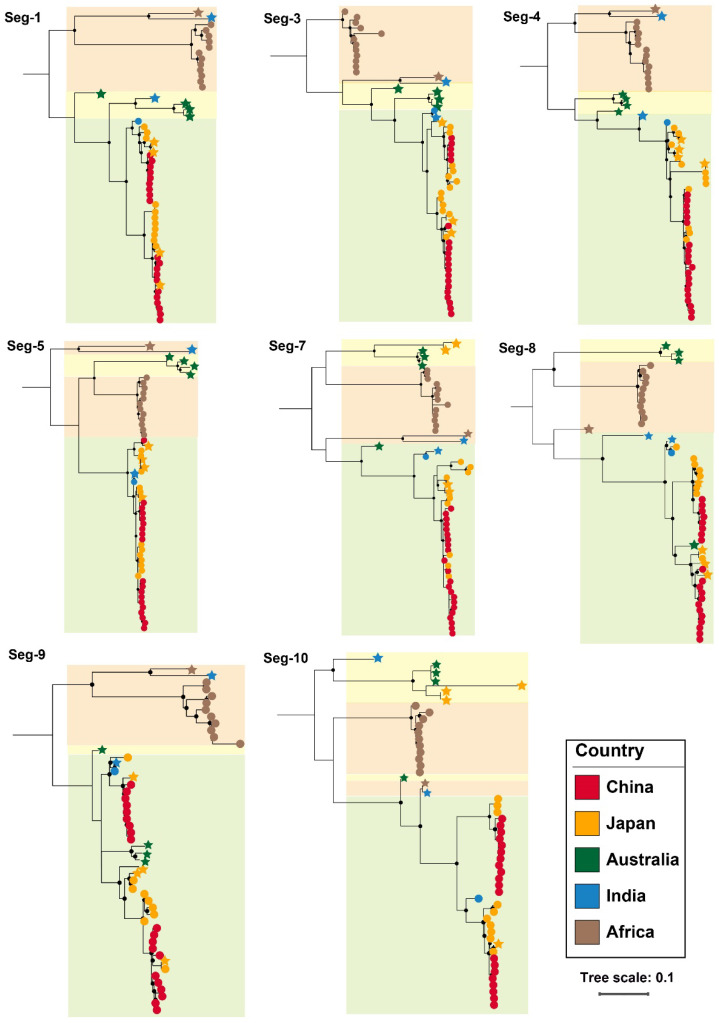
Maximum likelihood phylogenetic analysis of eight internal genomic segments of PALV. Maximum likelihood (ML) phylogenetic trees for segments encoding conserved core (Seg-1, -3, -4, -7, and -9) and non-structural proteins (Seg-5, -8 and -10). Nodal support with ultrafast bootstrap values ≥ 90% is indicated by solid black circles. Major continental clades—Asia (green background), Australia (yellow background), and Africa (beige background)—are highlighted to illustrate the global phylogeographic structure. Tip colors represent the country of origin: China (red), Japan (orange), Australia (dark green), India (blue), and Africa (brown). Strains identified as intercontinental reassortants are marked with a star (★), with their complete genomic constellations detailed in Figure S4 of the Supplementary Material.

Contrary to previous reports of a monophyletic Australian-Asian clade [4], phylogenetic analyses of eight PALV genome segments (Seg-1, -3 to -5, and -7 to -10) resolved global PALV sequences into three major continental lineages: Asia, Australia, and Africa, with bootstrap values exceeding 90% at the key nodes (Figure 4 and Figures S3–S10). However, this broad continental topology was disrupted by intercontinental reassortment among Australian, Asian, and African gene pools (Figure S11), providing direct evidence for the intercontinental transmission of the virus. Across the analyzed segments, Chinese and Japanese sequences clustered closely, with pairwise nucleotide identities ranging from 87.4% to 96.8%. For the majority of internal segments (Seg-1, Seg-3, Seg-5, and Seg-8 to 10), CHUV, BCV, and DAV isolates co-circulating in China and Japan did not segregate into serotype-specific monophyletic groups; instead, they formed heavily intertwined sublineages regardless of their outer capsid serotype designations. By contrast, Seg-4 and Seg-7 placed all Chinese isolates into a predominant cluster with a small number of Japanese isolates nested within it (Figure 4 and Figures S3–S10). This segment-to-segment phylogenetic inconsistency strongly hints at active genetic exchange between at least two sublineages co-circulating in both countries.

### 3.6. Screening and Detection of Intragenic Recombination Events Across PALV Genomic Segments

Comprehensive RDP5 screening across the global PALV genome alignments identified multiple putative intragenic recombination signals, which are compiled with their respective breakpoint coordinates and algorithmic support metrics in Supplementary Table S6. Within the East Asian viral population, a statistically robust intragenic recombination event was detected in Seg-3 of the Japanese BCV strain (ON-14/E/17). This recombinant locus involved the Japanese DAV strain (ON-3/E/17) as the putative major parent and the newly sequenced Chinese BCV strain (V256) as the minor parent (Table S6). This finding further corroborates, from the dimension of intragenic recombination, the active intra-regional genetic exchange among circulating strains in China and Japan. To guarantee the accuracy of temporal estimations, all genomic segments exhibiting evidence of recombination were strictly excluded from the subsequent Bayesian phylogeographic analyses.

### 3.7. Bayesian Evolutionary Timescale and Discrete Phylogeographic Reconstruction of PALV Lineages

**Figure 5:**
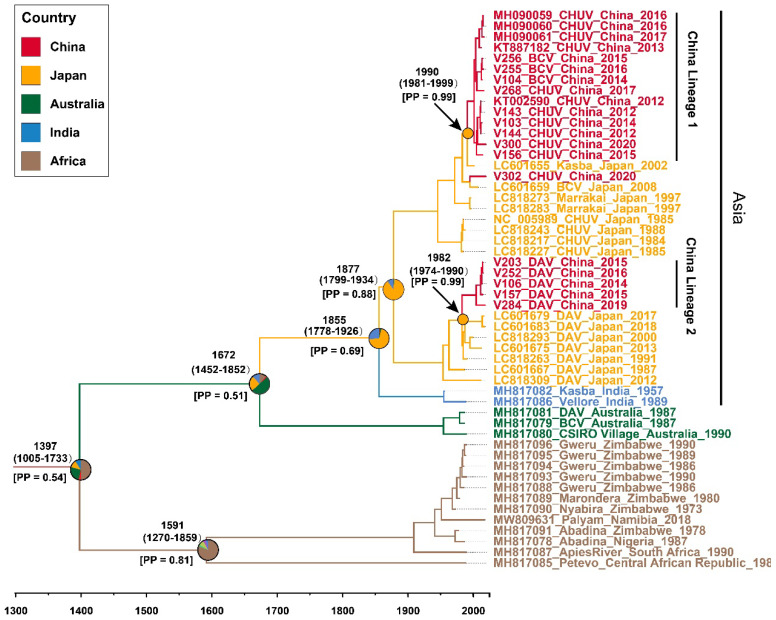
Spatiotemporal phylogeography of global PALV based on Seg-3 sequences. The maximum clade credibility (MCC) tree was inferred using a Bayesian MCMC approach. Branches are colored according to the most probable ancestral geographic location (see legend). Key nodes are annotated with the mean time to the most recent common ancestor (tMRCA) in CE, with 95% HPD intervals shown in parentheses. Pie charts at nodes represent the posterior probabilities of inferred ancestral locations. Arrows highlight the two inferred introductions from Japan to China.

To establish a temporal framework for PALV evolution, we evaluated the temporal signal strength of the non-recombinant gene segments. Excluding the hypervariable Seg-2 and Seg-6, the remaining eight segments exhibited clock-like signals suitable for time-scaled Bayesian analysis (R^2^ > 0.2), with mean evolutionary rates ranging from 2.70 to 3.84 × 10^−4^ substitutions/site/year (Table S7), which is comparable to the evolutionary rate of BTV [31]. Due to complex historical reassortment events (Figure S4), their global times to the most recent common ancestor (tMRCA) exhibited significant variation. Specifically, the mean ancestral divergence times spanned a historical range of over 600 years, dating from as early as 1032 CE (95% HPD: 547–1462) for Seg-1 to as recent as 1663 CE (95% HPD: 1481–1832) for Seg-9 (Table S7). As Seg-3 possessed the strongest temporal signal (R^2^ = 0.62), we selected this segment for high-resolution phylogeographic reconstruction. The time-scaled MCC tree (Figure 5) not only reproduced the three major geographic clades of Asia, Australia, and Africa, but more importantly, it reconstructed the transmission pathways within the Asian clade at an unprecedented resolution. Phylogeographic analysis explicitly indicated that Japan is the most probable source reservoir for the Asian PALV lineage (posterior probability [PP] = 0.69). From this Japanese source pool, PALV was introduced into China through at least two independent transmission events: an earlier introduction (Chinese Clade 2) occurred around 1982 CE (95% HPD: 1974–1990), while a more recent introduction (Chinese Clade 1) was traced back to approximately 1990 CE (95% HPD: 1981–1999). It is worth emphasizing that both of these geographic transition events from Japan to China were supported by extremely high posterior probabilities (PP = 0.99) (Figure 5).

## 4. Discussion

While the SNT is recognized as the gold standard for *Orbivirus* serotyping by the International Committee on Taxonomy of Viruses (ICTV), its utility is increasingly constrained by the scarcity of reference reagents [1]. To resolve the long-standing taxonomic ambiguity of PALV and provide a precise classification for our 15 Chinese isolates, this study established a sequence-based framework aligned with criteria used for major orbiviruses like BTV and EHDV [32,33]. By establishing a robust 73.7% VP2 amino acid identity threshold, we were able to unambiguously assign the newly isolated Chinese strains to the Chuzan, Bunyip Creek, and D’Aguilar genogroups. This rationalized framework not only resolves historical inconsistencies but also provides a scalable foundation for molecular diagnostics and epidemiological surveillance within China.

Although intragenic recombination is historically considered rare in segmented dsRNA viruses, evidence from BTV and AHSV identifies it as a significant, albeit infrequent, evolutionary mechanism [34,35]. The present study identifies multiple statistical signals of recombination in PALV. However, the high frequency of these detected signals warrants a cautious interpretation. We noted that the putative recombination events are disproportionately concentrated in strains originating from a single historical dataset [4]. From a technical perspective, chimeric sequences frequently emerge as computational artifacts during the *de novo* assembly of short-read NGS data [36]. Specifically, the recombination signal identified in the Segment 3 (VP3) of strain V256 was rigorously validated via Sanger sequencing, definitively ruling out assembly errors. This targeted validation suggests that true intragenic recombination does occur within the East Asian PALV population.

Genetic reassortment represents a hallmark evolutionary mechanism in segmented RNA viruses, facilitating the rapid emergence of novel genotypes with altered antigenicity or transmissibility, such as BTV and EHDV [37,38,39,40]. Beyond the macroscopic patterns of intercontinental reassortment, our findings emphasize that regional genetic exchange between China and Japan is a primary driver of PALV evolution in East Asia. The profound topological incongruence observed across different genomic segments of Chinese and Japanese isolates provides compelling evidence for ongoing transboundary dispersal and local reassortment. This pattern reinforces the concept of a unified “East Asian arbovirus ecosystem” where China and Japan act as interconnected nodes within a shared epidemiological network. Such active regional reassortment not only continuously reshapes the genetic diversity of circulating strains in southern China but also poses a potential risk for the emergence of novel variants with altered virulence or host range, necessitating sustained whole-genome-based surveillance in the region.

Our phylogeographic reconstruction elucidates the ancestral origin of Chinese PALV lineages, designating Japan as the primary source reservoir. Crucially, our analysis reveals frequent cross-border transmission between the two countries, as evidenced by the striking genetic affinity between the respective viral strains. This pattern of transboundary connectivity is not unique to PALV but exemplifies a broader regional epidemiological trend. Multiple Culicoides-borne viruses isolated in both China and Japan adhere to this pattern, including established transboundary pathogens (e.g., BTV, EHDV, and AKAV), as well as novel regional orbiviruses (e.g., Tibet orbivirus [TIBOV], Yunnan orbivirus [YOUV], and Guangxi orbivirus [GXOUV]) [8]. Collectively, this recurrent connectivity reinforces the hypothesis of a unified “East Asian arbovirus ecosystem,” wherein China and Japan operate not as isolated entities, but as interconnected nodes within a single epidemiological network.

Several concrete anthropogenic, climatic, and meteorological mechanisms may explain how this directional dispersal of PALV from Japan to China occurred. From an anthropogenic perspective, the 1980s and 1990s marked a transformative era of economic reform and opening-up in China, which triggered an intensive period of regional livestock trade and likely facilitated the introduction of PALV-infected live cattle into mainland China. From a climatic standpoint, although mainland Japan is generally perceived as a temperate zone, its southern regions (specifically the Kyushu and Okinawa archipelagos) share a humid subtropical climate with Southern China, maintaining year-round *Culicoides* vector activity and high viral endemicity that serve as a stable evolutionary reservoir. Critically, from a vector biology perspective, the transboundary movement of such orbiviruses is heavily governed by atmospheric dynamics rather than rigid latitudinal temperature gradients. During the peak summer and autumn arbovirus transmission seasons, the prevailing East Asian monsoon and powerful low-level jet streams, frequently augmented by seasonal typhoon tracks, generate highly viable atmospheric pathways. These macro-climatic phenomena act as an efficient airborne corridor, transporting infected *Culicoides* biting midges long distances across the East China Sea. Given these coupled environmental and anthropogenic drivers, this pronounced genetic affinity of circulating arboviruses between China and Japan strongly underscores the critical need for coordinated surveillance networks involving East Asian countries.

Our large-scale serological survey reveals the hyperendemic status of PALV in Chinese cattle (46.5% seroprevalence; 95% CI: 44.7–47.5%), characterized by a pronounced North–South dichotomy consistent with BTV and EHDV patterns [41,42]. This cross-sectional snapshot reveals a distinct geospatial stratification in PALV endemicity that closely aligns with regional variations in climate and elevation across the 15 surveyed provinces. Specifically, we identified a high-intensity transmission “corridor” (17.5° N–32.5° N, 97.5° E–115° E), within which infection risk shows a strong latitudinal dependence. This gradient reflects the fundamental ecological constraints of *Culicoides* vectors, which are notably inhibited by the cold, dry winters of the North but thrive in the warm, monsoon-dominated climate of the South. Crucially, the negligible seroprevalence (0.8%) observed in Tibet serves as a compelling ecological counter-evidence, where high-altitude cold limits vector survival despite its southern latitudinal position.

The hyperendemicity of PALV in southern China presents an epidemiological paradox: despite widespread viral circulation, overt clinical outbreaks remain virtually undocumented. We hypothesize that this discrepancy stems from a multifactorial interplay. First, the intrinsic pathogenicity of currently circulating strains may be inherently low, a phenomenon paralleling EHDV epidemiology in China, where endemic circulation often occurs without overt clinical disease [41]. Second, the high seroprevalence establishes the ecological foundation for “enzootic stability.” Under such a regime, widespread natural exposure likely confers protective immunity to heifers before sexual maturity, thereby safeguarding fetuses during subsequent gestations. Third, diagnostic oversight likely obscures the true disease burden. Due to a lack of clinical awareness, established pathogens such as BTV, EHDV, AKAV, Brucella, Bovine Viral Diarrhea (BVD), and Infectious Bovine Rhinotracheitis (IBR) are prioritized in current diagnostic frameworks for bovine reproductive disorders. Consequently, PALV is systematically excluded from differential diagnosis panels. Regardless of the primary driver, this widespread yet unmonitored transmission represents a “silent risk,” where viral evolution or stability disruption could trigger devastating outbreaks, necessitating active surveillance.

We recognize that the interpretation of our findings must be contextualized within the constraints inherent to our study design and data sources. First, our genetic analysis was based exclusively on PALV isolates from Yunnan and Guangdong provinces. Due to logistical constraints associated with maintaining long-term sentinel surveillance, the absence of isolates from other high-prevalence regions suggests that the genetic diversity and evolutionary complexity of Chinese PALV may be underestimated. Second, while our in-house c-ELISA proved robust for this large-scale survey, it was developed using whole CHUV virions. This virus was selected for assay development because of its established association with clinical disease in Japan [43] and its prevalence among our initial isolates. However, we acknowledge that next-generation assays using conserved recombinant proteins and monoclonal antibodies could provide improved standardization. Third, our phylogenetic reconstructions were constrained by the limited and biased sequence samples available in public databases. The scarcity of PALV genomes, particularly from India and Australia [4], likely introduced unavoidable uncertainty into the precise dating of ancestral nodes and inferred dispersal pathways.

In conclusion, the present study represents the first comprehensive characterization of PALV in China, redefining this virus from a neglected commensal into a hyperendemic “silent threat” within the East Asian Arbovirus Ecosystem. By integrating genomic and serological evidence, we not only elucidated a vector-driven transmission corridor in Southern China but also traced the viral origins to repeated introductions from Japan. Furthermore, our proposal to consolidate historical serotypes into seven sequence-based genogroups provides a robust framework for rationalizing global surveillance efforts. Ultimately, transitioning from passive observation to active risk assessment is imperative. Future research must prioritize resolving evolutionary blind spots by expanding genomic surveillance, experimentally quantifying the pathogenic potential of Chinese strains, and definitively identifying the specific *Culicoides* vector species. Such efforts are prerequisite for establishing a coordinated, transnational early-warning network capable of mitigating the emergence of PALV and related orbiviruses within the East Asian Arbovirus Ecosystem.

## Figures and Tables

**Table 1:** Regional distribution, physiographic characteristics, and sampling summary of the PALV serosurvey across 15 provinces in China, 2016–2018.

Region	Province	Longitude Range ° E	Latitude Range ° N	Elevation (m)	Climate Type	No. of Counties	No. of Groups	Total Samples	Sample Size per Group	Year
North	Inner Mongolia	97.2–126.0	37.4–53.3	900–1500	Semi-arid/Temperate	10 (2)	10 (2)	100	10	2018
	Hebei	113.1–119.8	36.1–42.6	0–1000	Temperate monsoon	6 (3)	6 (3)	295	20–100	2016–2018
Northwest	Xinjiang	73.6–96.4	34.3–49.2	400–1550	Arid continental	10 (2)	10 (2)	196	10–60	2018
Northeast	Jilin	121.6–131.3	40.8–46.3	100–500	Humid continental	3 (0)	3 (0)	120	20–50	2018
	Liaoning	119.2–125.8	38.7–43.5	0–500	Humid continental	3 (3)	3 (3)	150	50	2016
Central	Hubei	108.3–116.1	29.1–33.3	20–600	Humid subtropical	8 (8)	8 (8)	420	10–100	2017–2018
	Hunan	108.8–114.2	24.6–30.1	50–800	Humid subtropical	5 (5)	5 (5)	330	50–100	2016
Southwest	Tibet	78.4–99.1	26.8–36.5	3000–5000	Plateau alpine	26 (2)	26 (2)	355	5–60	2017
	Chongqing	105.2–110.2	28.2–32.3	200–1200	Humid subtropical	2 (2)	5 (5)	355	20–200	2017–2018
	Sichuan	97.4–108.5	26.0–34.3	500–3500	Subtropical monsoon/Alpine	5 (4)	5 (4)	77	10–25	2016
	Guizhou	103.6–109.5	24.6–29.2	500–1500	Humid subtropical	12 (8)	12 (8)	115	5–10	2016
	Yunnan	97.5–106.2	21.1–29.2	1000–3000	Subtropical highland	16 (16)	18 (18)	1678	48–360	2016–2018
South	Guangxi	104.5–112.1	20.9–26.4	0–600	South subtropical	9 (9)	9 (9)	152	10–40	2018
	Guangdong	109.7–117.3	20.2–25.5	0–500	South subtropical	3 (3)	3 (3)	52	16–20	2016
	Hainan	108.6–111.0	18.2–20.2	0–200	Tropical monsoon	4 (4)	6 (6)	380	40–100	2016–2017
Total	15 provinces	73.6–126.0	18.2–53.3	0–5000		119 (71)	129 (78)	4660	5–360	2016–2018

Data are presented as: Total count (Number of seropositive units in parentheses). A “sampling group” is defined as all samples collected from a single county within a specific calendar year. All 4660 serum samples were obtained from clinically healthy cattle (12–30 months of age) with no history of PALV vaccination. Elevation and climate types represent the characteristic features of each province.

**Table 2:** Geographic and climatic characteristics of the five sentinel surveillance sites in Yunnan and Guangdong Provinces, China.

Site	Province	Longitude	Latitude	Elevation (m)	Mean Annual Temperature (°C)	Mean Annual Precipitation (mm)
Shizhong	Yunnan	103.98	24.82	1850	13.5–14.5	1100–1300
Jinghong	Yunnan	100.80	22.02	580	21.5–22.5	1200–1400
Jiangcheng	Yunnan	101.87	22.58	1120	18.0–19.0	2200–2400
Mangshi	Yunnan	98.58	24.43	920	19.5–20.5	1650–1850
Shantou	Guangdong	116.68	23.35	5	21.0–22.0	1500–1700

Mean annual temperature and precipitation data were obtained from local meteorological stations and represent the average values recorded during the sentinel surveillance period (2012–2020). Elevation values correspond to the specific GPS coordinates of the sentinel herds.

**Table 3:** Summary of sampling efforts for PALV isolation and serological survey.

Purpose	Time Period	Sample Type	Sampling Site (Province)	Total Samples	Key Results
Virus isolation	2012–2017	Sentinel animal blood samples	Yunnan & Guangdong	2340	25 PALV isolates
	2019–2020		Yunnan	156	4 PALV isolates
Serological Survey	2016–2018	Bovine serum samples	15 provinces of China	4660	2167 positive samples

**Table 4:** Seroprevalence and associated odds ratios of PALV infection across 15 provinces in China, 2016–2018.

Region	Province	Seroprevalence % (n/N)				95% CI (%)	Adjusted Odds Ratio (95% CI)	*p*-Value
		2016	2017	2018	Total			
North	Inner Mongolia	NA	NA	8.0 (8/100)	8.0 (8/100)	3.5–15.0	Referent	NA
	Hebei	2.0 (2/100)	1.1 (1/95)	1.0 (1/100)	1.4 (4/295)	0.5–3.5	0.2 (0.1–0.5)	0.011
Northeast	Jilin	NA	NA	0.0 (0/120)	0.0 (0/120)	0.0–3.6	0.1 (0.0–0.1)	0.007
	Liaoning	4.0 (6/150)	NA	NA	4.0 (6/150)	1.7–8.5	0.5 (0.2–1.4)	0.184
Northwest	Xinjiang	NA	NA	4.6 (9/196)	4.6 (9/196)	2.4–8.6	0.6 (0.2–1.5)	0.231
Central	Hubei	NA	68.0 (136/200)	55.0 (121/220)	61.2 (257/420)	56.6–65.6	17.0 (8.6–38.4)	<0.001
	Hunan	39.4 (130/330)	NA	NA	39.4 (130/330)	34.1–44.7	7.5 (3.5–15.9)	<0.001
Southwest	Tibet	NA	0.8 (2/240)	NA	0.8 (2/240)	0.2–3.0	0.1 (0.0–0.5)	0.004
	Guizhou	33.0 (38/115)	NA	NA	33.0 (38/115)	25.0–42.2	5.7 (2.5–12.9)	<0.001
	Chongqing	NA	46.8 (103/220)	33.3 (45/135)	41.7 (148/355)	36.6–47.0	8.2 (4.0–16.9)	<0.001
	Sichuan	74.0 (57/77)	NA	NA	74.0 (57/77)	63.2–82.7	32.8 (13.5–79.2)	<0.001
	Yunnan	30.6 (110/360)	61.2 (347/567)	82.8 (622/751)	64.3 (1079/1678)	60.9–65.6	19.8 (9.8–40.2)	<0.001
South	Guangxi	NA	NA	88.8 (135/152)	88.8 (135/152)	82.9–92.9	89.5 (37.9–220.0)	<0.001
	Guangdong	71.2 (37/52)	NA	NA	71.2 (37/52)	57.4–81.9	28.2 (11.1–72.6)	<0.001
	Hainan	69.5 (139/200)	65.6 (118/180)	NA	67.6 (257/380)	62.9–72.3	24.0 (11.3–51.1)	<0.001
Total	15 provinces	37.5 (519/1384)	47.1 (707/1502)	53.0 (941/1774)	46.5 (2167/4660)	44.7–47.5	10.2 (5.1–20.3)	<0.001

Data are presented as Seroprevalence % (n/N), where ‘n’ is the number of positive samples and ‘N’ is the total number of samples tested. Abbreviations: NA, not available; CI, confidence interval. The adjusted Odds Ratio and 95% CIs were derived from a multivariable logistic regression model with the province of Inner Mongolia selected as the reference category due to its highest seroprevalence among northern provinces. Statistical significance was defined as a *p*-value of less than 0.05.

**Table 5:** Characteristics of 15 representative PALV isolates from sentinel cattle in southern China selected for whole-genome sequencing.

Serotype	Isolate	Origin (County, Province)	Date Isolated	Host Species	Passage History	GenBank Acc. No.
CHUV	V144	Jiangcheng, Yunnan	2012-05-24	Cattle (calf)	C6/36 (p1), BHK-21 (p3)	PX290519-PX290528
CHUV	V143	Shizhong, Yunnan	2012-09-07	Cattle (calf)	C6/36 (p2), BHK-21 (p5)	PX334463-PX334472
CHUV	V103	Shizhong, Yunnan	2014-07-17	Cattle (calf)	C6/36 (p1), BHK-21 (p4)	PX334473-PX334482
CHUV	V156	Mangshi, Yunnan	2015-10-11	Cattle (calf)	C6/36 (p1), BHK-21 (p2)	PX290529-PX290538
CHUV	V268	Mangshi, Yunnan	2017-06-05	Cattle (calf)	C6/36 (p2), BHK-21 (p4)	PX334483-PX334492
CHUV	V300	Jinghong, Yunnan	2020-07-18	Water buffalo	C6/36 (p1), BHK-21 (p4)	PX290479-PX290488
CHUV	V302	Jiangcheng, Yunnan	2020-07-03	Cattle (calf)	C6/36 (p1), BHK-21 (p3)	PX334493-PX334502
BCV	V104	Shizhong, Yunnan	2014-09-21	Cattle (calf)	C6/36 (p1), BHK-21 (p3)	PX290499-PX290508
BCV	V256	Shantou, Guangdong	2015-07-11	Cattle (cow)	C6/36 (p2), BHK-21 (p3)	PX290489-PX290498
BCV	V255	Mangshi, Yunnan	2016-06-28	Cattle (calf)	C6/36 (p1), BHK-21 (p3)	PX290469-PX290478
DAV	V252	Shantou, Guangdong	2016-06-10	Cattle (cow)	C6/36 (p3), BHK-21 (p2)	PX334503-PX334512
DAV	V106	Shizhong, Yunnan	2014-07-08	Cattle (calf)	C6/36 (p1), BHK-21 (p4)	PX334513-PX334522
DAV	V157	Jiangcheng, Yunnan	2015-09-19	Cattle (calf)	C6/36 (p1), BHK-21 (p2)	PX290509-PX290518
DAV	V203	Shizhong, Yunnan	2015-07-23	Cattle (calf)	C6/36 (p1), BHK-21 (p4)	PX334523-PX334532
DAV	V284	Jinghong, Yunnan	2019-05-05	Water buffalo	C6/36 (p1), BHK-21 (p5)	PX334533-PX334542

Passage history indicates the cell lines used and the number of serial passages (p) in Aedes albopictus (C6/36) and Baby Hamster Kidney (BHK-21) cells. GenBank accession numbers provided represent the range for all ten genome segments (Seg-1 to Seg-10) of each isolate. Abbreviations: CHUV, Chuzan virus; BCV, Bunyip Creek virus; DAV, D’Aguilar virus.

**Table 6:** Proposed classification of Palyam virus (PALV) into seven VP2 genogroups and their relationship with historical serotypes.

Genogroups	Historical Serotype/Virus Name	Temporal Range	Geographic Distribution	No. of Seg-2 Sequences (n)
Chuzan	Chuzan/Kasba virus	1957–2020	**China**, Japan, India	23
	Vellore virus	1989	India	1
	Abadina virus	1978–1990	Nigeria, Zimbabwe	3
Gweru	Gweru virus	1986–1990	Zimbabwe	5
	CSIRO Village virus	1990	Australia	1
D’Aguilar	D’Aguilar virus	1972–2020	**China**, Japan, Australia	17
	Palyam virus	1992, 2018	India, Namibia	2
	Nyabira virus	1973, 1990	Zimbabwe	3
Marondera	Marondera virus	1985	Central African Republic	1
	Apies River virus	1990	South Africa	1
Bunyip Creek	Bunyip Creek virus	1976–2020	**China**, Japan, Australia	11
Marrakai	Marrakai virus	1989,1997	Australia, Japan	3
Petevo	Petevo virus	1989	Central African Republic	1

Genogroups were defined as monophyletic lineages with ultrafast bootstrap support >90% in the maximum-likelihood phylogenetic analysis of Seg-2 sequences. The seven-genogroup framework is supported by a VP2 amino acid identity threshold value of 73.7% for genogroup demarcation. The total number of sequences (*n* = 72) includes both archival sequences and the 15 new Chinese isolates from this study. For emphasis, ‘China’ is bolded and underlined in the table.

## Data Availability

The viral genome sequences generated and analyzed during the current study have been deposited in the GenBank database under the accession numbers provided in Table S6. All other datasets supporting the conclusions of this article, including the detailed results of the large-scale serosurvey, are available from the corresponding authors upon reasonable request.

## Supplementary Materials

The following supporting information can be downloaded at: https://www.mdpi.com/article/10.3390/v18060638/s1, Figure S1: Morphological characterization of purified CHUV particles through transmission electron microscope; Figure S2: Phylogenetic analysis and sequence identity of global PALV Seg-6/VP5 sequences; Figures S3–S10: phylogenies for Seg-1, Seg-3, Seg-4, Seg-5, Seg-7, Seg-8, Seg-9, and Seg-10 of global PALV strains; Figure S11: Genomic composition of PALV strains exhibiting intercontinental reassortment. Table S1: Statistical formulas and definitions used for seroprevalence calculations; Table S2: Primers and probes for group and type-specific detection of PALV by RT-PCR and qRT-PCR; Table S3: Marginal likelihood estimation for model selection using Seg-3; Table S4: MCMC convergence diagnostics and posterior summary statistics for the BEAST analysis of PALV genome segments; Table S5: Next-generation sequencing data output and quality control statistics for the 15 newly sequenced PALV genomes; Table S6: Putative intragenic recombination events detected in the PALV genomes; Table S7: Best-fit substitution models, evolutionary rates, and tMRCA estimates for PALV genome segments. Text S1: Detailed development, optimization, and standardized protocol of the PALV competitive ELISA (c-ELISA).
