# Supplementary material for: Genomic Epidemiology and National Seroprevalence Reveal the Widespread Distribution of Palyam Viruses in China
Source: Viruses. 2026 Jun 3;18(6):638. doi: 10.3390/v18060638 (PMC13307812; doi:10.3390/v18060638)
Supplement: Supplementary file 1 [file viruses-18-00638-s001.zip › viruses-4265875-supplementary.pdf]

Table S1

Table S1. Statistical formulas and definitions used for seroprevalence calculations.

Indicator	Definition / Formula	Statistical Method
Individual-level Seroprevalence	$P_{ind} = (n/N) \times 100$ (n: number of samples with PI ≥ 50.0%; N: total serum samples)	Descriptive statistics
Group-level Seroprevalence	$Pg = (g/G) \times 100$ (g: number of positive county-year groups; G: total groups)	Spatial unit analysis
Equivocal Results	Samples with PI values between 45.0% and 50.0%	Re-tested once; excluded if remaining equivocal
95% Wilson Confidence Interval (CI)	Used for calculating the precision of prevalence estimates	Wilson score interval
Seropositive Group Definition	A group is positive if it contains at least one sample with PI ≥ 50.0%	Binary classification

Table S2

Table S2. Primers and probes for group and type-specific detection of PALV by RT-PCR and qRT-PCR

Target gene	Primer and probe name	Primer sequence (5′-3′)[a]	Nucleotide location	Product size (bp)	Description of primers and probes
Seg-7	PALV-Seg7-F<br>PALV-Seg7-R	GCCAACTACACAACAAGAAAG<br>CCGAATATAACAGTAAAGCCA	171-192<br>920-941	791	Pan-RT-PCR primers of PALV
Seg-2	CHUV-Seg2-F<br>CHUV-Seg2-R	GGATTCACGCYATTACTCGGA<br>ATGGATTTCTTGTTGGGATTAT	1175-1195<br>2157-2178	1004	Serotype-specific RT-PCR primers of CHUV
Seg-2	BCV-Seg2-F<br>BCV-Seg2-R	GRGCTRTCATAGATTCGGAAGC<br>TACAAACYTTTACYAGAGCCAT	965–987<br>2215-2237	1251	Seroytpe-specific RT-PCR primers of BCV
Seg-2	DAV-Seg2-F<br>DAV-Seg2-R	ATTTTTCAACGAAGAGACGTG<br>ATAGGAACACCTTTACCYAGG	929-949<br>2131-2151	1223	Serotype-specific RT-PCR primers of DAV
Seg-7	PALV-S7/YG-F<br>PALV-S7/YG-R<br>PLAV-S7-Probe	CATCAATGGCAACAATCGGTG<br>ATTCAGCATACCTGTAATTCGTAC<br>FAM-TTCCATATACAACGTCGGCAATGACAAG-BHQ1	275-295<br>346-369<br>314-341	94	Pan-qRT-PCR primers and probe of PALV
Seg-2	CHUV-S2/YG-F<br>CHUV-S2/YG-R<br>CHUV-S2-Probe	TGCGTGGGACCATTGATAGAG<br>GTGAATCAACCGCCCAAACTC<br>FAM-ATGCACCGACCCCACTCACTGACG-BHQ1	1969-1989<br>2049-2069<br>2018-2041	101	Serotype-specific qRT-PCR primers and probe of CHUV
Seg-2	BCV-S2/YG-F<br>BCV-S2/YG-R<br>BCV-S2-Probe	GTACATGATTTTCCGCAACTATC<br>TCCTCTATTTTGATCCGAAACATA<br>FAM-CTCTTCGGCTTATTCCATTCYTCATCTC-BHQ1	1667-1689<br>1774-1797<br>1734-1761	131	Serotype-specific qRT-PCR primers and probe of BCV
Seg-2	DAV-S2/YG-F<br>DAV-S2/YG-R<br>DAV-S2-Probe	GGAGGAGTGTCGAAGAGCAC<br>GTGCTGCCACTCTGTYTCAT<br>FAM-TAGYTGAATATAAACGTGCGGGYCG-BHQ1	1583-1602<br>1692-1711<br>1647-1671	129	Serotype-specific qRT-PCR primers and probe of DAV

Table Notes: TaqMan probes were labeled with carboxyfluorescein (FAM) at their 5'-end and black hole quencher (BHQ1) at their 3'-end.

Table S3

Table S4 . Marginal likelihood estimation for model selection using Seg-3.

Molecular Clock Model	Coalescent Tree Prior	Log Marginal Likelihood (PS)	Log Marginal Likelihood (SS)	ΔlnL (Relative to best)	Rank
UCLN	Bayesian Skyline	-13,763	-13,757	0	1
UCLN	Exponential	-13,771	-13,758	-1.00	2
UCLN	Constant Size	-13,775	-13,765	-8.00	3
Strict Clock	Exponential	-13,778	-13,771	-14.00	4
Strict Clock	Constant Size	-13,781	-13,774	-17.00	5
Strict Clock	Bayesian Skyline	-13,776	-13,776	-19.00	6

Table Notes: Marginal likelihoods were estimated using both Path Sampling (PS) and Stepping Stone (SS) methods with 20 steps and 5,000,000 generations per step. R^2 for Seg-3 was 0.62. The combination of an Uncorrelated Lognormal Relaxed (UCLN) clock and a Bayesian Skyline coalescent prior yielded the highest marginal likelihood. While the likelihood difference between Rank 1 and Rank 2 was marginal (ΔlnL = 1.0), the Bayesian Skyline model was selected for its flexibility in capturing non-parametric demographic fluctuations. This optimized framework was subsequently applied to all segments with $R^2 > 0.2$.

Table S4

Table S5. MCMC convergence diagnostics and posterior summary statistics for the BEAST analysis of PALV genome segments.

Segment	Mean prior [ESS]	Mean Likelihood [ESS]	Mean Tree Height (tMRCA) [ESS]	Mean ucld.mean [ESS]	Result
Seg-1	-3175 [475]	-19521 [4583]	1032 [505]	2.696 E-4 [491]	Pass
Seg-3	-3158 [1300]	-12098 [11556]	1302 [1094]	3.539 E-4 [1308]	Pass
Seg-4	-3026 [1050]	-10553 [3536]	1351 [1029]	3.237 E-4 [1113]	Pass
Seg-5	-3018 [2354]	-5147 [6727]	1596 [1689]	3.006 E-4 [2585]	Pass
Seg-7	-3174 [1417]	-4861 [6050]	1339 [1668]	2.712 E-4 [1532]	Pass
Seg-8	-3113 [926]	-4868 [5407]	1377 [1101]	3.835 E-4 [916]	Pass
Seg-9	-3120 [1910]	-3601 [6642]	1663 [2110]	3.755 E-4 [1972]	Pass
Seg-10	-3132 [1557]	-3328 [7512]	1276 [1686]	3.579 E-4 [1725]	Pass

Table Notes: Three independent MCMC chains were run for 100 million generations per segment, sampling every 10,000 generations. Statistics were calculated after a 10% burn-in using Tracer v1.7.27. Mean Tree Height represents the estimated time (in years) from the most recent sampling point back to the root of the tree. The corresponding calendar years (CE) are reported in Table 4 in the main text. ESS, Effective Sample Size. "Pass" denotes that all primary parameters—including posterior likelihood, prior, tree height, and substitution rates (ucld.mean)—achieved an ESS > 200, indicating robust sampling and convergence of the independent MCMC chains . Segments (Seg-2 and Seg-6) with $R^2 < 0.2$ in the TempEst analysis were excluded from this Bayesian inference to ensure temporal signal reliability.

Table S5

Table S7. Data output and quality control statistics for the 15 newly sequenced PALV genomes.

Virus strain	Raw Reads(bp)	Clean Reads(bp)	GC(%)	Q20(%)	Q30(%)
V144	1,428,265,500	790,023,300	44.13	95.38	89.11
V143	1,552,726,800	849,331,200	44.30	95.39	89.08
V103	1,503,978,000	671,341,800	46.65	93.98	86.41
V156	1,577,373,600	794,001,000	43.32	95.05	88.38
V268	1,554,471,300	817,230,300	43.88	95.05	88.40
V300	1,549,353,300	815,954,700	40.02	95.54	89.52
V302	1,752,254,400	921,577,200	42.76	95.55	89.40
V104	1,759,464,300	929,081,700	45.03	95.00	88.26
V256	1,700,674,500	872,813,400	43.79	95.03	88.35
V255	1,650,832,500	839,286,000	43.59	95.46	89.17
V252	1,802,088,900	935,840,100	44.25	95.22	88.70
V106	1,674,101,400	1,586,295,400	42.92	97.32	92.77
V157	1,751,897,400	1,660,337,700	43.04	97.40	92.91
V203	1,674,065,700	1,590,681,600	44.21	97.39	92.94
V284	2,238,303,000	2,101,943,900	44.74	97.45	93.31

Table Notes: The table summarizes the next-generation sequencing metrics for each viral isolate. Raw Reads: total number of reads generated; Clean Reads: number of reads remaining after quality filtering; GC(%): percentage of guanine and cytosine bases in the sequence; Q20(%): percentage of bases with a Phred quality score > 20 (indicating ≥ 99% base call accuracy); Q30(%): percentage of bases with a Phred quality score > 30 (indicating ≥ 99.9% base call accuracy).

Table S6

Table S6. Putative intragenic recombination events detected in the PALV genomes

Recombinant Strain Virus (strain) (Country)	Segment	Breakpoints	Major Parent Virus (strain) (Country)	Minor Parent Virus (strain) (Country)	Supported Methods
Vellore virus (68886) (IND)	Seg-1	838–2023	Kasba virus (GG15534) (IND)	CSIRO Village virus (CSIRO11) (AUS)	7
	Seg-1	2108–2714	Kasba virus (GG15534) (IND)	Gweru virus (1832/79) (ZIM)	7
	Seg-1	3198–3860	Kasba virus (GG15534) (IND)	DAV B8112 (AUS)	7
	Seg-4	1263–1899	CSIRO Village virus (CSIRO11) (AUS)	Kasba virus (GG15534) (IND)	5
Nyabira virus (1772/74) (ZIM)	Seg-2	2005–2467	Nyabira (792/73) (ZIM)	Unknown	6
	Seg-3	1118–1237	Nyabira (792/73) (ZIM)	Unknown	4
	Seg-3	1701–1778	Nyabira (792/73) (ZIM)	Unknown	4
DAV (B8112) (AUS)	Seg-5	1460–1630	BCV (CSIRO87) (AUS)	Nyabira (1772/74) (ZIM)	7
	Seg-6	997–1076	Nyabira (1772/74) (ZIM)	Marondera virus (1070/78) (ZIM)	6
CSIRO Village virus (CSIRO11) (AUS)	Seg-6	317–584	Unknown	Gweru virus (1828/82) (ZIM)	4
	Seg-6	833–1562	Unknown	Gweru (1856/76) (ZIM)	6
Palyam virus DVTD (IND)	Seg-2	1108–1252	DAV (B8112) (AUS)	Unknown	6
	Seg-8	400–707	Petevo (ARB2032) (CAF)	Kasba virus (GG15534) (IND)	6
BCV (ON-14/E/17) (JPN)	Seg-3	1942–2668	DAV (ON-3/E/17) (JPN)	BCV (V256) (CHN)	5

Table Notes: Breakpoints indicate the nucleotide positions of recombination sites within the sequence alignment. Each row represents an independent recombination event. Multiple entries for a single segment (e.g., Seg-1 of Vellore virus) denote distinct events with different breakpoints or parental origins. "Supported Methods" refers to the number of algorithms (out of seven: RDP, GENECONV, BootScan, MaxChi, Chimaera, SiScan, and 3Seq) that simultaneously detected and validated the recombination event with a significance threshold of $p < 0.05$. All genomic alignments were screened using the RDP5 software package. The recombination event in Seg-3 of the Japanese strain BCV (ON-14/E/17) involving the Chinese isolate V256 was independently validated by Sanger sequencing. Abbreviations: IND, India; AUS, Australia; ZIM, Zimbabwe; JPN, Japan; CAF, Central African Republic; CHN, China.The detailed GenBank accession numbers and metadata for all global reference strains involved in these events are comprehensively compiled in the newly added Supplementary Table S8

Table S7

Table S7. Best-fit substitution models, evolutionary rates, and tMRCA estimates for PALV genome segments.

Segment	No. of sequences	Best-fit model (BIC)	Temporal Signal (R^2)	Estimated tMRCA (CE)		Substitution rate	
				Mean	95% HPD	Mean	95% HPD
Seg-1	53	TIM2+F+G4	0.54	1032	547-1462	2.70	1.78-3.60
Seg-2	52	TIM3+F+I+G4	3.26×10^{-2}	NA	NA	NA	NA
Seg-3	52	TIM2+F+G4	0.62	1302	865-1681	3.39	2.07-4.79
Seg-4	55	TIM2+F+I+G4	0.57	1351	1119-1558	3.24	2.40-4.13
Seg-5	49	TIM2+F+G4	0.37	1596	1282-1871	3.07	1.96-4.37
Seg-6	54	TIM2+F+I+G4	0.17	NA	NA	NA	NA
Seg-7	53	K3Pu+F+G4	0.62	1339	1005-1644	2.71	1.70-3.70
Seg-8	50	TIM2+F+G4	0.43	1377	945-1734	3.84	2.20-5.70
Seg-9	51	TN+F+G4	0.33	1663	1481-1832	3.76	2.35-5.28
Seg-10	51	TN+F+G4	0.24	1276	860-1614	3.58	2.14-5.15

Table Notes: R^2, coefficient of determination from TempEst analysis. Segments with $R^2 < 0.2$ were considered to have an insufficient temporal signal for reliable evolutionary rate and time-scale estimation and were therefore excluded from the BEAST analysis. tMRCA, time to the most recent common ancestor. CE, Common Era. 95% HPD, 95% highest posterior density interval. Substitution rates are expressed in 10^{-4} substitutions/site/year. NA, not applicable.BIC, Bayesian Information Criterion**.** The best-fit nucleotide substitution models were selected based on the minimum BIC values using IQtree v2.0.

Figure S1

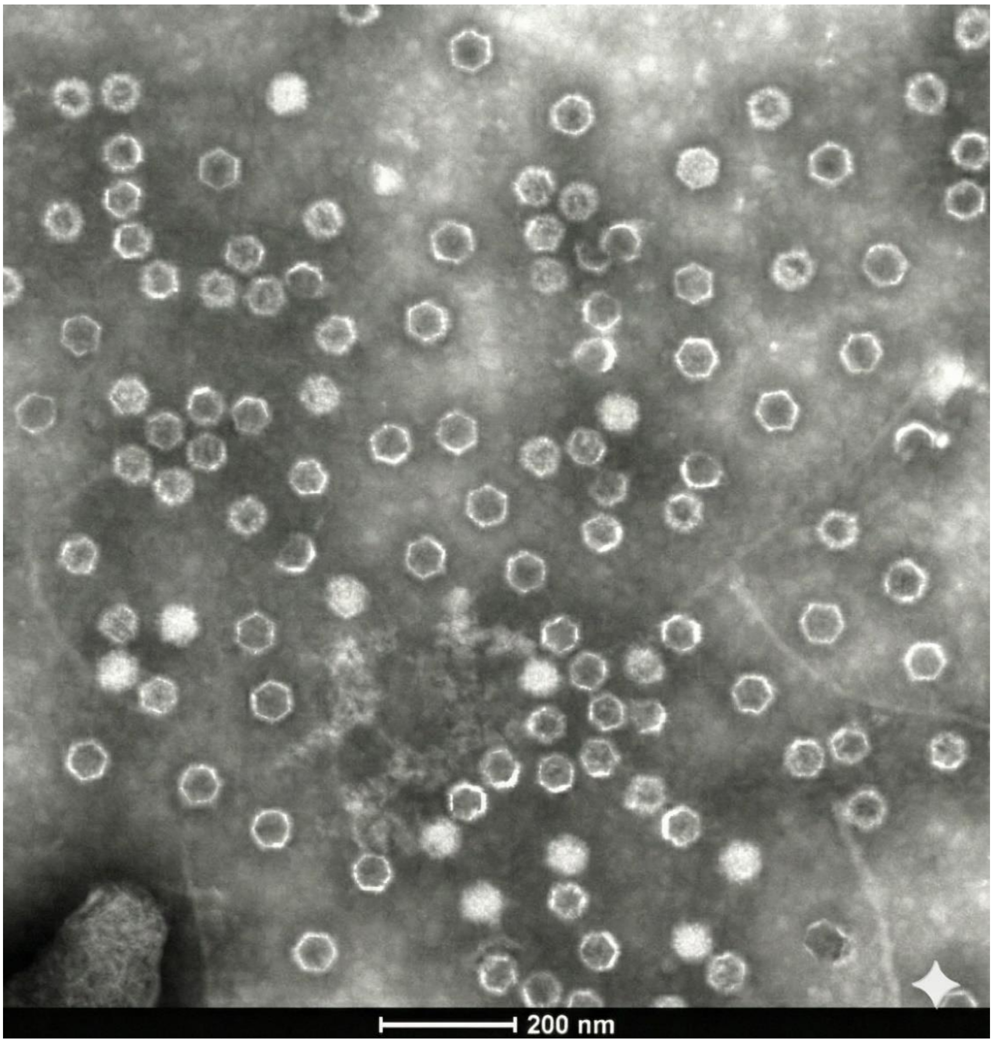

Figure S1. Morphological characterization of purified Chuzan virus (CHUV) particles. Transmission electron micrograph of purified CHUV particles after negative staining with 2% phosphotungstic acid. Observations were performed using a JEM-1400 electron microscope (JEOL Ltd., Akishima, Tokyo, Japan). The viral particles exhibit typical orbivirus-like morphology with a consistent diameter of approximately 75–80 nm. The preparation shows high homogeneity and purity, with no visible host-cell contamination, confirming its suitability for ELISA plate coating.. Scale bar = 200 nm.

Figure S2

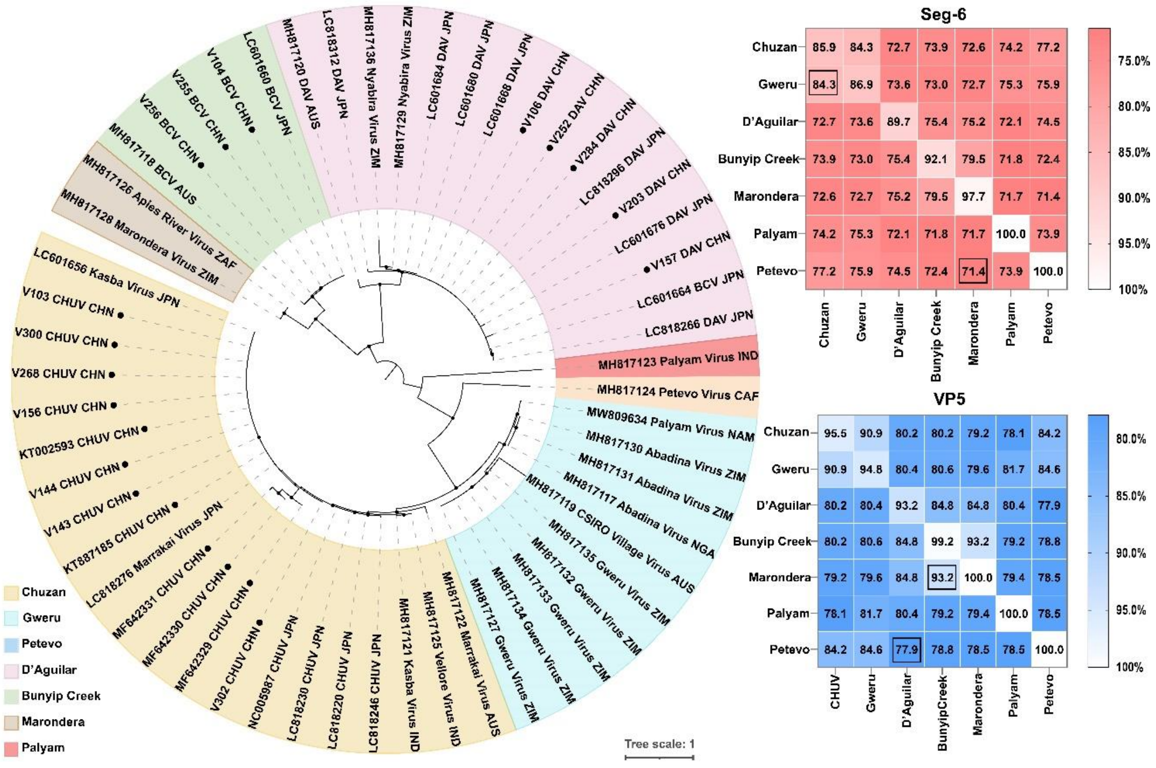

Figure S2. Phylogenetic analysis and sequence identity of global PALV Seg-6/VP5 sequences

Left: A maximum likelihood (ML) tree was inferred from VP5 amino acid sequences. Nodal support ⩾90% is indicated by black circles. Strains are labeled as 'Accession number_Virus name_Country code'. The seven VP5 sequence-based groups are highlighted. Chinese isolates are marked with black circles (●). **Right:** Heatmaps display pairwise sequence identities. The upper matrix shows nucleotide identities (Seg-6); the lower shows amino acid identities (VP5). Diagonal values represent minimum intra-group identities; off-diagonal values represent maximum inter-group identities.

Figure S3

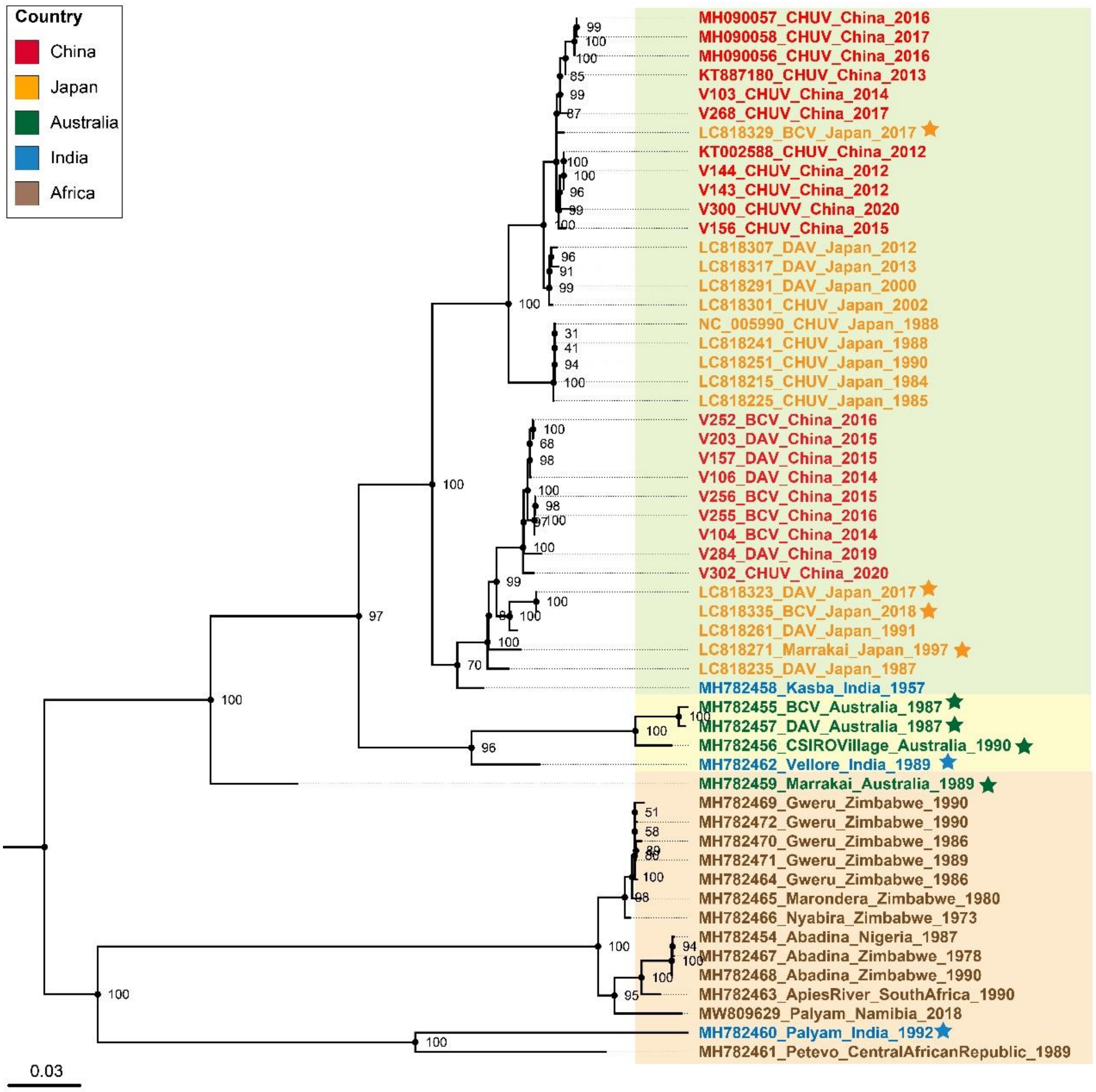

Figure S3. Maximum-likelihood phylogenetic analysis of Palyam virus genomic Segment 1 (Seg-1).

The tree was inferred under the best-fit TIM2+F+G4 nucleotide substitution model using IQ-TREE v2.0 with 10,000 ultrafast bootstrap replicates. Numerical values at the nodes represent ultrafast bootstrap support percentages. Major continental clades—Asia (green background), Australia (yellow background), and Africa (beige background)—are highlighted to illustrate the global phylogeographic structure. Tip text colors represent the country of origin: China (red), Japan (orange), Australia (dark green), India (blue), and Africa (brown). Strains identified as intercontinental reassortants are marked with colored stars (★) corresponding to their respective countries, with their mosaic genomic constellations detailed in Supplementary Figure S4. The scale bar at the bottom left indicates 0.03 nucleotide substitutions per site.

Figure S4

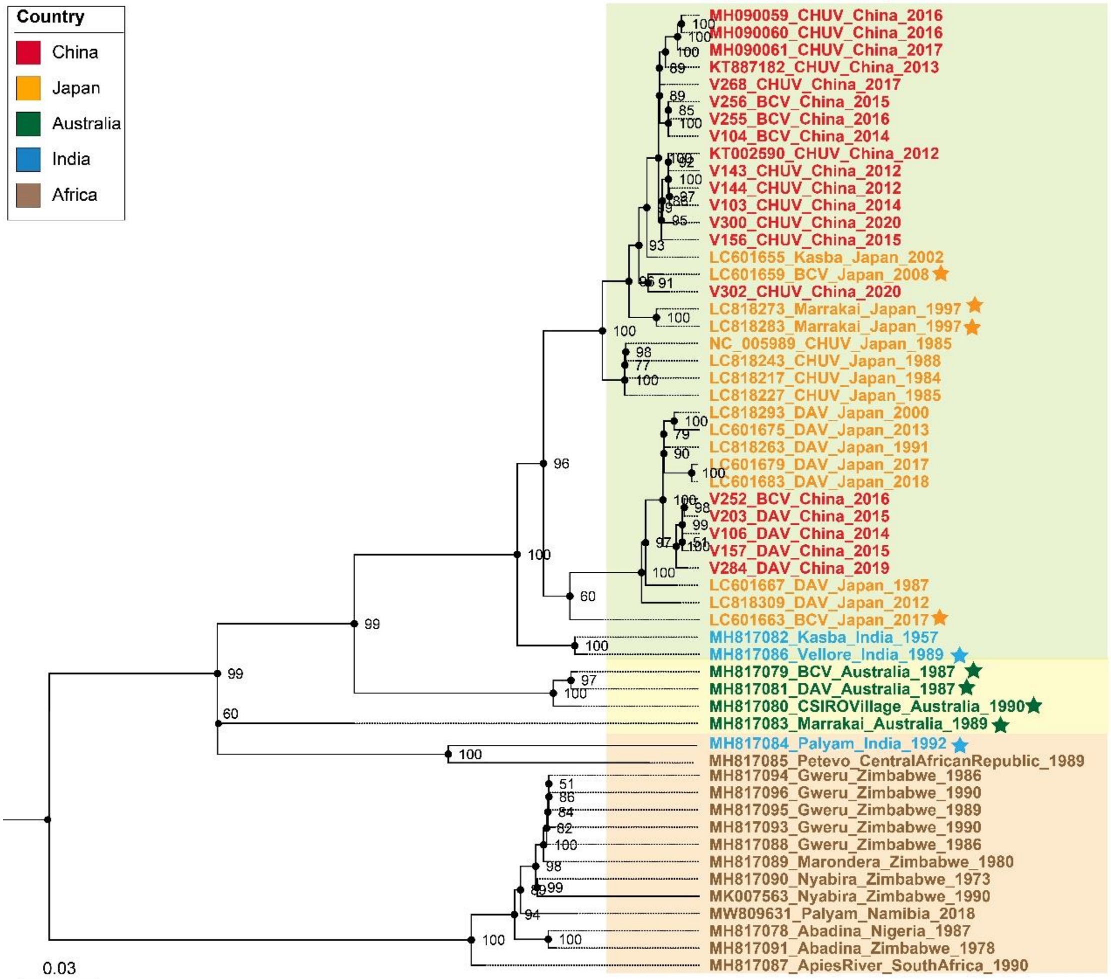

Figure S4. Maximum-likelihood phylogenetic analysis of Palyam virus genomic Segment 3 (Seg-3).

The tree was inferred under the best-fit TIM2+F+G4 nucleotide substitution model using IQ-TREE v2.0 with 10,000 ultrafast bootstrap replicates. Numerical values at the nodes represent ultrafast bootstrap support percentages. Major continental clades—Asia (green background), Australia (yellow background), and Africa (beige background)—are highlighted to illustrate the global phylogeographic structure. Tip text colors represent the country of origin: China (red), Japan (orange), Australia (dark green), India (blue), and Africa (brown). Strains identified as intercontinental reassortants are marked with colored stars (★) corresponding to their respective countries, with their mosaic genomic constellations detailed in Supplementary Figure S4. The scale bar at the bottom left indicates 0.03 nucleotide substitutions per site.

Figure S5

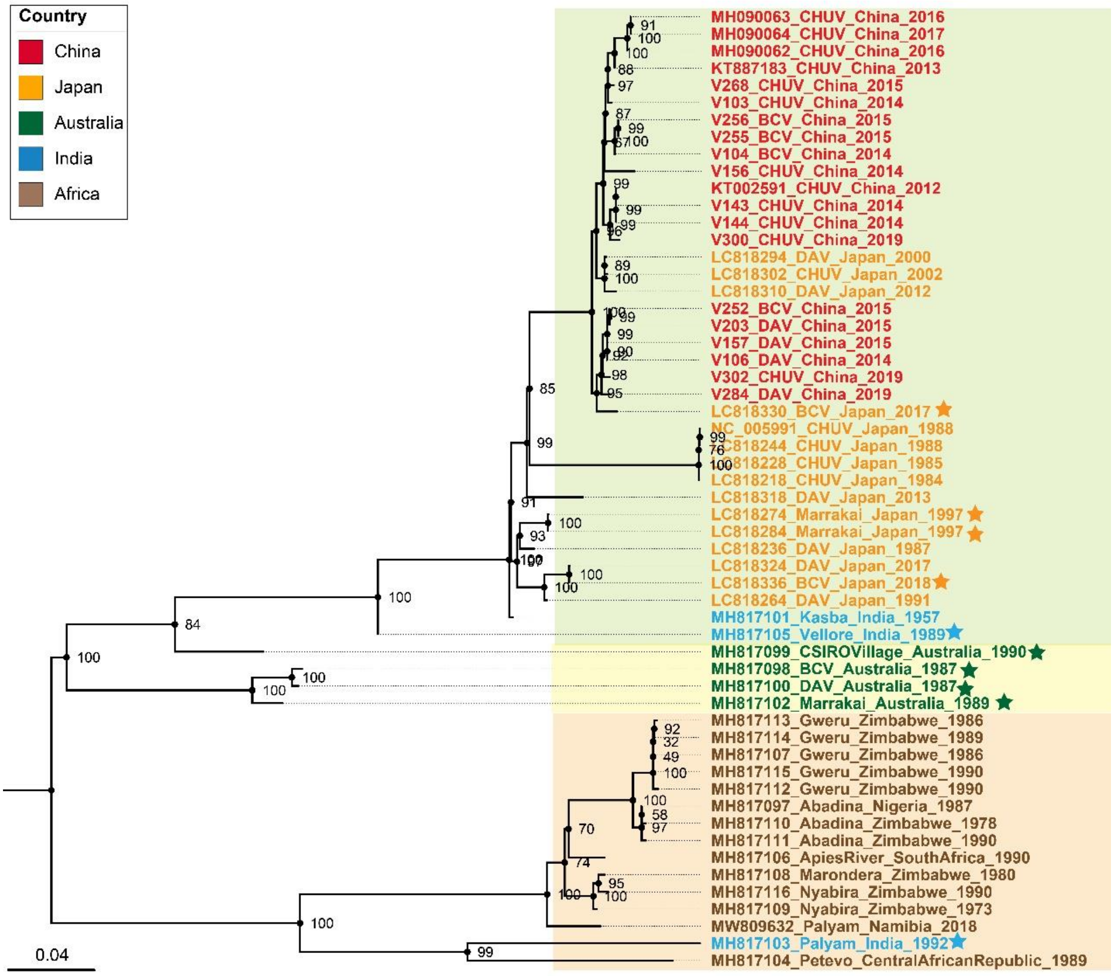

Figure S5. Maximum-likelihood phylogenetic analysis of Palyam virus genomic Segment 4 (Seg-4).

The tree was inferred under the best-fit K3Pu+F+G4 nucleotide substitution model using IQ-TREE v2.0 with 10,000 ultrafast bootstrap replicates. Numerical values at the nodes represent ultrafast bootstrap support percentages. Major continental clades—Asia (green background), Australia (yellow background), and Africa (beige background)—are highlighted to illustrate the global phylogeographic structure. Tip text colors represent the country of origin: China (red), Japan (orange), Australia (dark green), India (blue), and Africa (brown). Strains identified as intercontinental reassortants are marked with colored stars (★) corresponding to their respective countries, with their mosaic genomic constellations detailed in Supplementary Figure S4. The scale bar at the bottom left indicates 0.03 nucleotide substitutions per site.

Figure S6

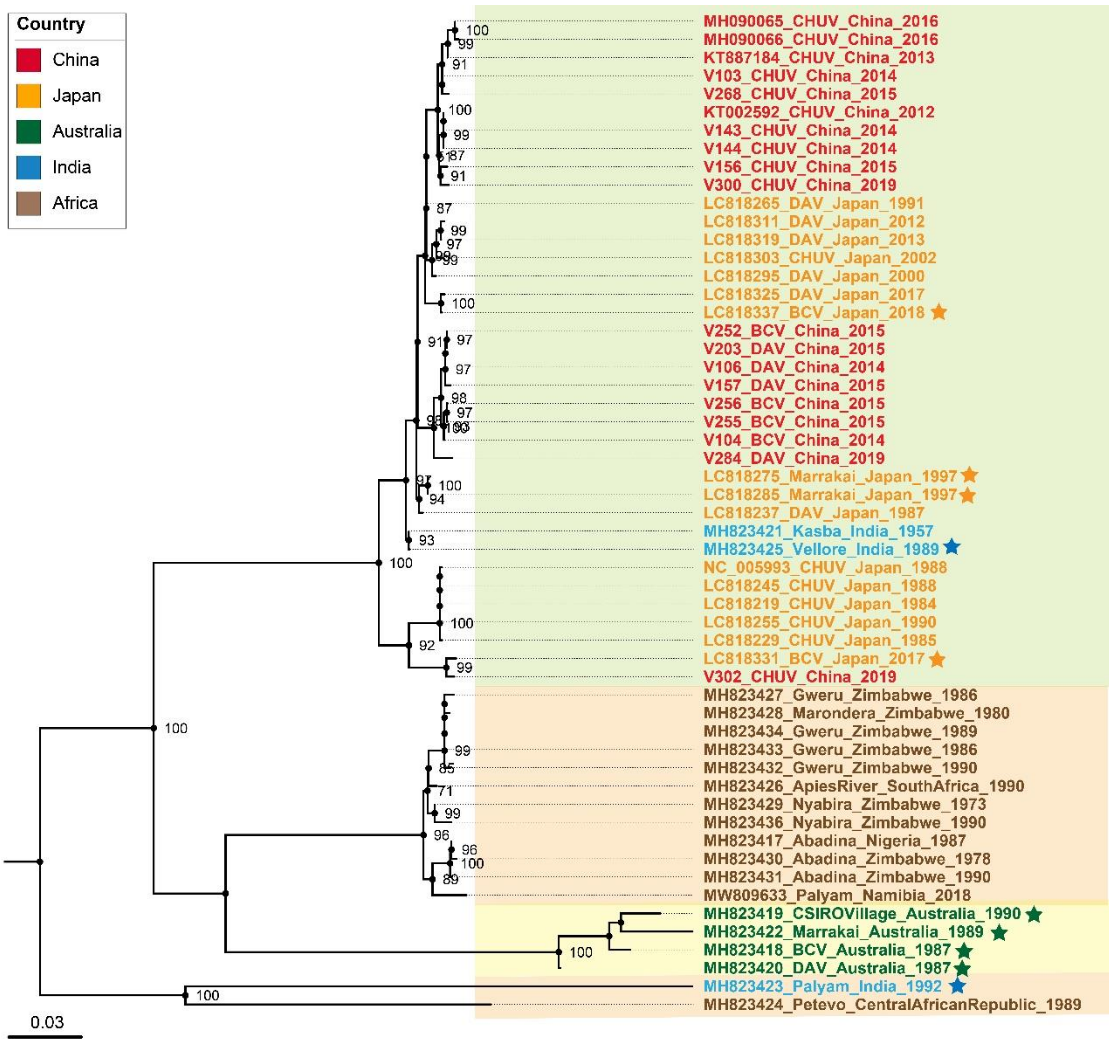

Figure S6. Maximum-likelihood phylogenetic analysis of Palyam virus genomic Segment 5 (Seg-5).

The tree was inferred under the best-fit TIM2+F+G4 nucleotide substitution model using IQ-TREE v2.0 with 10,000 ultrafast bootstrap replicates. Numerical values at the nodes represent ultrafast bootstrap support percentages. Major continental clades—Asia (green background), Australia (yellow background), and Africa (beige background)—are highlighted to illustrate the global phylogeographic structure. Tip text colors represent the country of origin: China (red), Japan (orange), Australia (dark green), India (blue), and Africa (brown). Strains identified as intercontinental reassortants are marked with colored stars (★) corresponding to their respective countries, with their mosaic genomic constellations detailed in Supplementary Figure S4. The scale bar at the bottom left indicates 0.03 nucleotide substitutions per site.

Figure S7

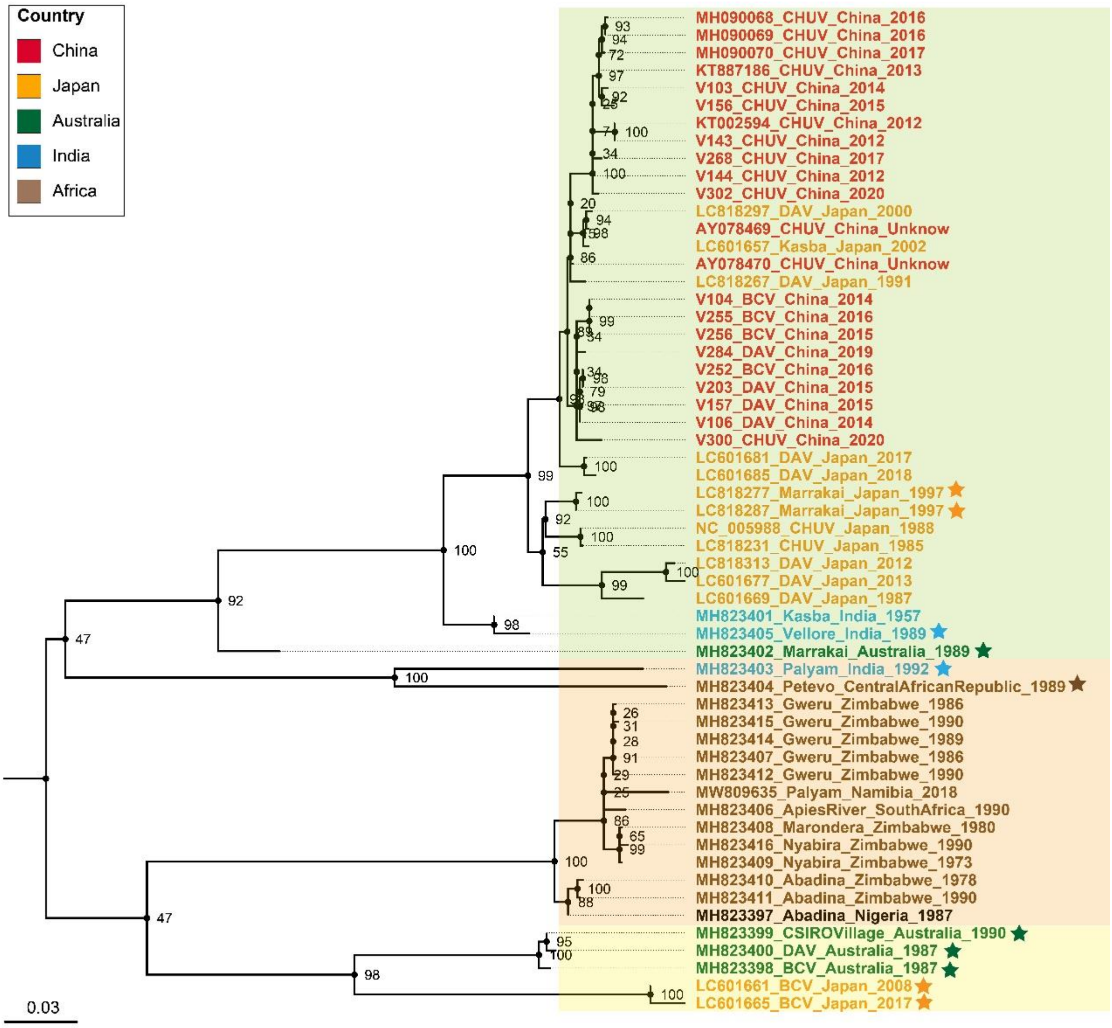

Figure S7. Maximum-likelihood phylogenetic analysis of Palyam virus genomic Segment 7 (Seg-7).

The tree was inferred under the best-fit TIM2+F+G4 nucleotide substitution model using IQ-TREE v2.0 with 10,000 ultrafast bootstrap replicates. Numerical values at the nodes represent ultrafast bootstrap support percentages. Major continental clades—Asia (green background), Australia (yellow background), and Africa (beige background)—are highlighted to illustrate the global phylogeographic structure. Tip text colors represent the country of origin: China (red), Japan (orange), Australia (dark green), India (blue), and Africa (brown). Strains identified as intercontinental reassortants are marked with colored stars (★) corresponding to their respective countries, with their mosaic genomic constellations detailed in Supplementary Figure S4. The scale bar at the bottom left indicates 0.03 nucleotide substitutions per site.

Figure S8

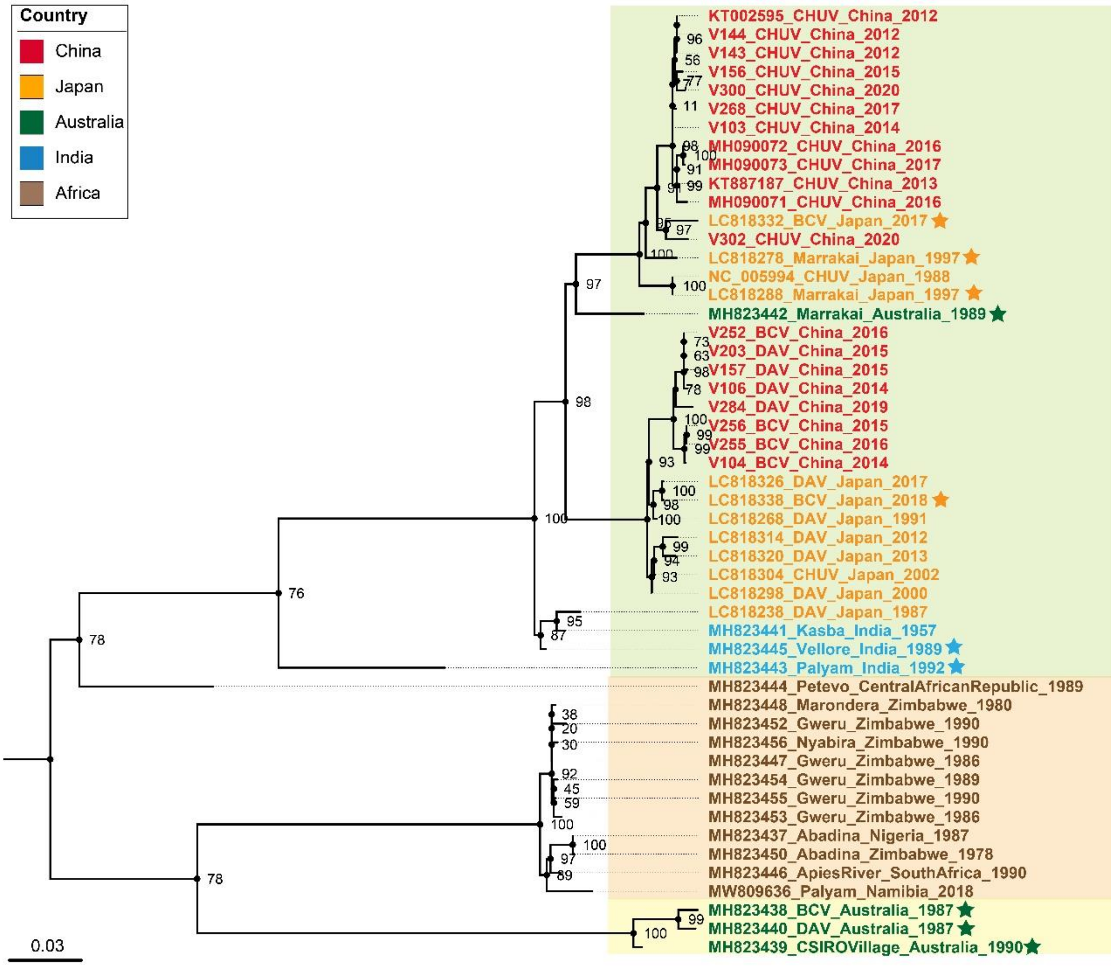

Figure S8. Maximum-likelihood phylogenetic analysis of Palyam virus genomic Segment 8 (Seg-8).

The tree was inferred under the best-fit TIM2+F+G4 nucleotide substitution model using IQ-TREE v2.0 with 10,000 ultrafast bootstrap replicates. Numerical values at the nodes represent ultrafast bootstrap support percentages. Major continental clades—Asia (green background), Australia (yellow background), and Africa (beige background)—are highlighted to illustrate the global phylogeographic structure. Tip text colors represent the country of origin: China (red), Japan (orange), Australia (dark green), India (blue), and Africa (brown). Strains identified as intercontinental reassortants are marked with colored stars (★) corresponding to their respective countries, with their mosaic genomic constellations detailed in Supplementary Figure S4. The scale bar at the bottom left indicates 0.03 nucleotide substitutions per site.

Figure S9

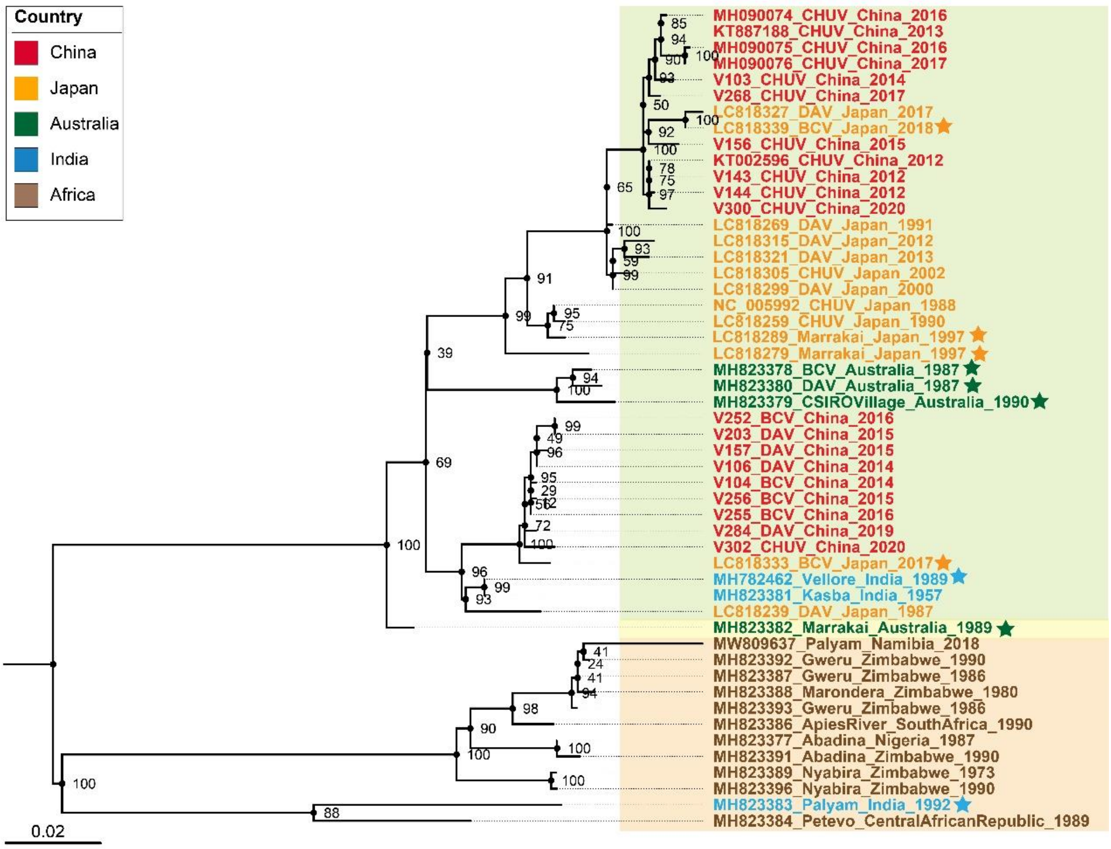

Figure S9. Maximum-likelihood phylogenetic analysis of Palyam virus genomic Segment 9 (Seg-9).

The tree was inferred under the best-fit TIM2+F+G4 nucleotide substitution model using IQ-TREE v2.0 with 10,000 ultrafast bootstrap replicates. Numerical values at the nodes represent ultrafast bootstrap support percentages. Major continental clades—Asia (green background), Australia (yellow background), and Africa (beige background)—are highlighted to illustrate the global phylogeographic structure. Tip text colors represent the country of origin: China (red), Japan (orange), Australia (dark green), India (blue), and Africa (brown). Strains identified as intercontinental reassortants are marked with colored stars (★) corresponding to their respective countries, with their mosaic genomic constellations detailed in Supplementary Figure S4. The scale bar at the bottom left indicates 0.03 nucleotide substitutions per site.

Figure S10

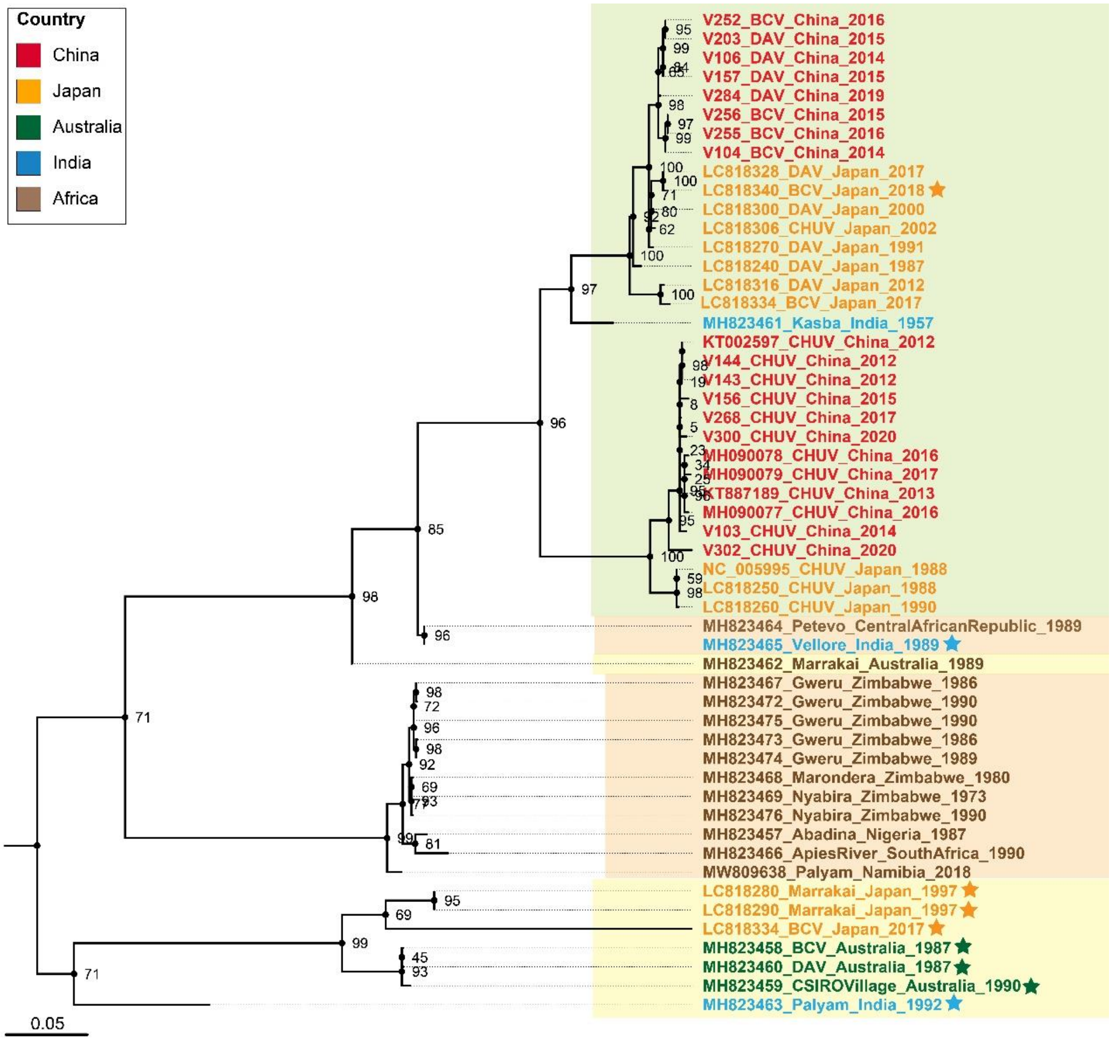

Figure S10. Maximum-likelihood phylogenetic analysis of Palyam virus genomic Segment 10 (Seg-10).

The tree was inferred under the best-fit TIM2+F+G4 nucleotide substitution model using IQ-TREE v2.0 with 10,000 ultrafast bootstrap replicates. Numerical values at the nodes represent ultrafast bootstrap support percentages. Major continental clades—Asia (green background), Australia (yellow background), and Africa (beige background)—are highlighted to illustrate the global phylogeographic structure. Tip text colors represent the country of origin: China (red), Japan (orange), Australia (dark green), India (blue), and Africa (brown). Strains identified as intercontinental reassortants are marked with colored stars (★) corresponding to their respective countries, with their mosaic genomic constellations detailed in Supplementary Figure S4. The scale bar at the bottom left indicates 0.03 nucleotide substitutions per site.

Figure S11

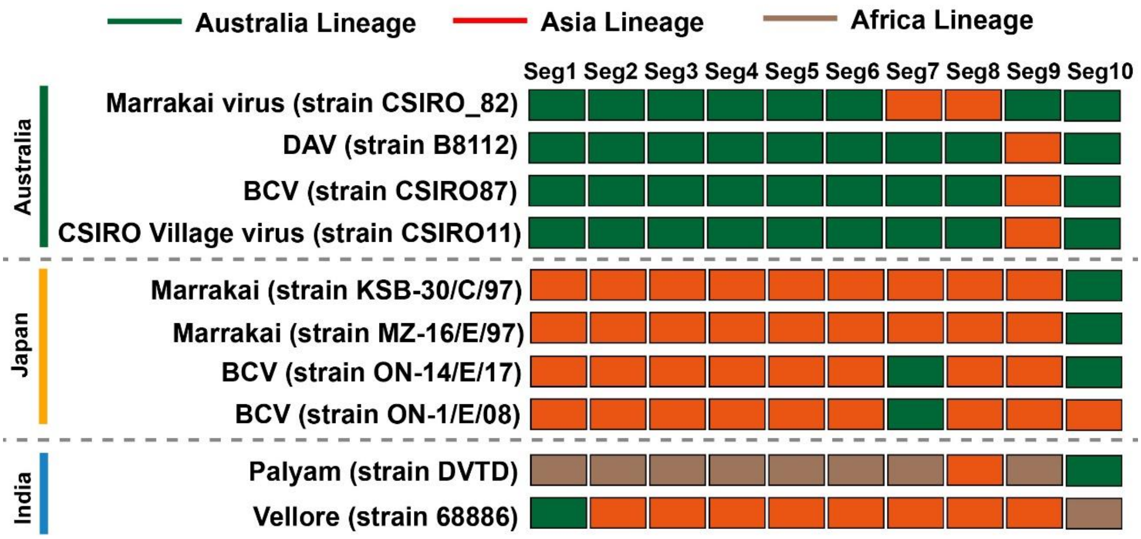

Figure S11. Genomic composition of PALV strains exhibiting intercontinental reassortment.

Each row represents a viral strain, grouped by geographic origin. Colors indicate the continental lineage assigned to each segment via Maximum-likelihood phylogenetic analysis: Australia (green), Asia (orange), and Africa (brown). Dashed lines separate the major geographic groups. The mosaic genomic constellations of Indian strains highlight the role of the Indian subcontinent as a geographical bridge for intercontinental PALV dispersal.

Text S1

Text S1. Detailed development, optimization, and standardized protocol of the PALV competitive ELISA (c-ELISA).

1. Antigen Preparation and Characterization

（1）**Virus Propagation**: The plaque-purified CHUV (strain V144) was propagated in C6/36 cells cultured in MEM supplemented with 2% FBS. Cells were infected at a multiplicity of infection (MOI) of 0.1. When the cytopathic effect (CPE) reached approximately 70%, the infected cells were harvested. To release the intracellular virions, the cell suspension underwent three consecutive freeze-thaw cycles.

（2）**Virus Purification and Quantification**: The cell lysate was first clarified by centrifugation at 12,000 × g for 15 min at 4°C to remove cell debris. The resulting supernatant was then layered over a 30% (w/v) sucrose cushion and subjected to ultracentrifugation at 140,000 × g for 3 h at 4°C using an SW 32 Ti rotor (Beckman Coulter). The viral pellet was gently resuspended in phosphate-buffered saline (PBS, pH 7.4). The total protein concentration of the purified virions was quantified using a BCA Protein Assay Kit (Takara Bio Inc., Shiga, Japan) following the manufacturer's instructions.

（3）**Morphological Characterization of purified virion:** To verify the integrity and purity of the purified CHUV particles, the viral suspension was examined by negative staining transmission electron microscopy (TEM). Briefly, 10 μL of the purified antigen was carbon-coated onto copper grids and stained with 2% phosphotungstic acid (pH 7.0). Observations were performed using an electron microscope. The TEM analysis revealed homogenous, spherical particles with a diameter of approximately 75–80 nm, displaying the characteristic morphology of *Orbiviruses*. No significant host-cell contamination was observed, confirming the suitability of the preparation for ELISA plate coating.

2. Competitive Antibody Production and Optimization

（1）**Immunization**: Guinea pigs were immunized three times at two-week intervals with 50 μg of β-propiolactone-inactivated CHUV virions emulsified in Freund's complete adjuvant (Sigma-Aldrich, St. Louis, MO, USA). Two booster injections were administered using the same antigen dose emulsified in Freund's incomplete adjuvant. Complete virus inactivation was confirmed by three blind passages in BHK-21 cells, showing an absence of cytopathic effect (CPE).

（2）**IgG Purification**: Hyperimmune sera were pooled and total IgG was purified using a two-step procedure: initial enrichment via 40% saturated ammonium sulfate precipitation, followed by fine purification using Protein G affinity chromatography (Cytiva, Marlborough, MA, USA). The purified IgG was dialyzed against PBS (pH 7.4).

3. Plate Coating and Blocking Procedure

（1）**Coating Procedure**: High-binding Nunc MaxiSorp 96-well microplates (Thermo Fisher Scientific, Waltham, MA, USA) were used. Purified CHUV antigen was diluted to 4.12 μg/mL in 0.05 M carbonate–bicarbonate buffer (pH 9.6), and 100 μL of the mixture was added to each well. The plates were sealed with adhesive film and incubated overnight (12–16 h) at 4°C.

（2）**Blocking**: Following incubation, the coating solution was discarded, and the plates were washed three times with PBST as described in Section 4. Then, each well was blocked with 200 μL of blocking buffer (5% skim milk in PBST) and incubated for 1 h at 37°C. After blocking, the plates were washed again three times before serum addition.

4. Assay Optimization and Quality Control

（1）**Checkerboard Titration and Optimization:** The optimal working concentrations of the coating antigen and the competitive IgG were determined by checkerboard titration. The selection criteria were: (i) the OD_{450} of the negative control OD_{neg} was approximately 1.5; and (ii) the Signal-to-Noise (S/N) ratio (calculated as OD_{neg}/OD_{pos} using a strong positive reference serum) was > 10. Based on these criteria, an antigen concentration of 4.12 μg/mL and a competitive IgG dilution of 1:6,000 were selected.

（2） **Internal Quality Control:** Each plate included duplicate wells of known PALV-seronegative and PALV-seropositive bovine sera. The mean OD_{450} of the negative controls, OD_{neg} served as the reference value for PI calculations. To ensure assay validity, the mean OD_{neg} was required to be **between 1.0 and 2.0**, and the PI of the positive control had to be > 50%. If these conditions were not met, the assay was repeated.

（3）**Diagnostic Validation and Reference Serum Panel:** The diagnostic threshold (PI ≥ 50.0%) and performance metrics (93.3% sensitivity and 97.3% specificity) were established using a reference panel of 250 bovine sera (including CHUV, BCV, and DAV genogroups), as detailed in Section 2.3 of the main text.

5. Standardized c-ELISA Protocol

（1）**Washing Protocol**: Washing was performed using PBS containing 0.05% Tween-20 (PBST). Plates were washed three times between each successive step, with a 30–60 s soak period per cycle. Residual liquid was removed by inverting and tapping onto absorbent paper towels.

（2）**Sequential Competition**: 50 μL of test serum (1:20) was added and incubated at 37°C for 1 h. After washing the plates three times with PBST, 50 μL of purified guinea pig anti-CHUV IgG (1:6,000) was added directly to the wells and incubated for an additional 1 h at 37°C.

（3）**Secondary Antibody**: 100 μL of HRP-conjugated goat anti-guinea pig IgG (Abcam, Cambridge, MA, USA), diluted 1:5,000 in blocking buffer, was added and incubated for 1 h at 37°C.

（4）**Substrate and Termination**: 100 μL of TMB substrate (Sigma-Aldrich) was added and incubated in the dark for 15 min at room temperature. The reaction was terminated by adding 50 μL of 2 M H_2SO_4.

（5）**Measurement**: Optical density (OD) was measured at 450 nm using a Multiskan FC microplate reader (Thermo Fisher Scientific, Waltham, MA, USA)
